# LncRNA-HGBC stabilized by HuR promotes gallbladder cancer progression by regulating miR-502-3p/SET/AKT axis

**DOI:** 10.1186/s12943-019-1097-9

**Published:** 2019-11-21

**Authors:** Yun-ping Hu, Yun-peng Jin, Xiang-song Wu, Yang Yang, Yong-sheng Li, Huai-feng Li, Shan-shan Xiang, Xiao-ling Song, Lin Jiang, Yi-jian Zhang, Wen Huang, Shi-li Chen, Fa-tao Liu, Chen Chen, Qin Zhu, Hong-zhuan Chen, Rong Shao, Ying-bin Liu

**Affiliations:** 1 Department of General Surgery, Xinhua Hospital, Affiliated to Shanghai Jiao Tong University School of Medicine, Building 25, Room 513, 1665 Kongjiang Road, Shanghai, 200092 China; 2 Shanghai Key Laboratory of Biliary Tract Disease Research, 1665 Kongjiang Road, Shanghai, 200092 China; 3 Shanghai Research Center of Biliary Tract Disease, 1665 Kongjiang Road, Shanghai, 200092 China; 4 Department of Pharmacology, Shanghai Jiao Tong University School of Medicine, W. Building 3, Room 407, 280 Chongqi Road, Shanghai, 200025 China

**Keywords:** Gallbladder cancer, lncRNA-HGBC, miR-502-3p, SET, HuR

## Abstract

**Backgrounds:**

Long non-coding RNAs (lncRNAs) are essential factors that regulate tumor development and metastasis via diverse molecular mechanisms in a broad type of cancers. However, the pathological roles of lncRNAs in gallbladder carcinoma (GBC) remain largely unknown. Here we discovered a novel lncRNA termed lncRNA Highly expressed in GBC (lncRNA-HGBC) which was upregulated in GBC tissue and aimed to investigate its role and regulatory mechanism in the development and progression of GBC.

**Methods:**

The expression level of lncRNA-HGBC in GBC tissue and different cell lines was determined by quantitative real-time PCR. The full length of lncRNA-HGBC was obtained by 5′ and 3′ rapid amplification of the cDNA ends (RACE). Cellular localization of lncRNA-HGBC was detected by fluorescence in situ hybridization (FISH) assays and subcellular fractionation assay. In vitro and in vivo assays were preformed to explore the biological effects of lncRNA-HGBC in GBC cells. RNA pull-down assay, mass spectrometry, and RNA immunoprecipitation (RIP) assay were used to identify lncRNA-HGBC-interacting proteins. Dual luciferase reporter assays, AGO2-RIP, and MS2-RIP assays were performed to verify the interaction between lncRNA-HGBC and miR-502-3p.

**Results:**

We found that lncRNA-HGBC was upregulated in GBC and its upregulation could predict poor survival. Overexpression or knockdown of lncRNA-HGBC in GBC cell lines resulted in increased or decreased, respectively, cell proliferation and invasion in vitro and in xenografted tumors. LncRNA-HGBC specifically bound to RNA binding protein Hu Antigen R (HuR) that in turn stabilized lncRNA-HGBC. LncRNA-HGBC functioned as a competitive endogenous RNA to bind to miR-502-3p that inhibits target gene SET. Overexpression, knockdown or mutation of lncRNA-HGBC altered the inhibitory effects of miR-502-3p on SET expression and downstream activation of AKT. Clinically, lncRNA-HGBC expression was negatively correlated with miR-502-3p, but positively correlated with SET and HuR in GBC tissue.

**Conclusions:**

Our study demonstrates that lncRNA-HGBC promotes GBC metastasis via activation of the miR-502-3p-SET-AKT cascade, pointing to lncRNA-HGBC as a new prognostic predictor and a therapeutic target.

## Introduction

Gallbladder cancer (GBC) is a rare, but is an extremely aggressive carcinoma developed from the biliary tract [1]. Regardless of the relatively higher incidence in Asia than in Western countries [2], almost GBC cases are diagnosed at advanced stages due to the lack of early symptoms. Thus, most of those patients fall into unexpected contraindication of the resection, while the rest need immediately surgical removal [3–5]. To date, adjunctive therapy including chemo- and radiotherapy is an indispensable regimen to treat the majority of GBC patients. However, the efficacy of those conventional therapies is transient and barely minimal, as most of the patients rapidly recur and concomitantly develop chemo- and radio-resistance [6]. Hence, the overall prognosis of GBC is extraordinarily poor and the mean survival ranges from 13.2 to 19 months [7, 8]. Although over the last decades large effort has been made in identification of tumor-promoting oncogenes and tumor suppressors in GBC, there is still lack of independent biomarkers that can be routinely used in clinical practice [9, 10]. Therefore, it is of paramount importance to identify novel factors that potentially serve as novel diagnostic biomarkers and therapeutic targets for the treatment of patients with GBC.

Long non-coding RNAs (lncRNAs) comprise a wide variety of ncRNA species with a minimum length of 200 nucleotides(nt) but deficiency of protein-coding potential [11–13]. LncRNAs share many characteristics with mRNAs in which they are transcribed by RNA polymerase II and can be capped, spliced, and polyadenylated. LncRNAs are located in intergenic DNA, introns, or overlapping with other genes in an antisense orientation, and their expression is usually restricted to specific tissue. Emerging lines of evidence have shown that lncRNAs are involved in diverse biological processes and are key regulators in pathologic process at both transcriptional and post-transcriptional level [14–16]. It is noted that, a new regulatory mechanism termed competing endogenous RNAs (ceRNAs) has been identified to mediate lncRNA activity. In this model, lncRNA or mRNA harboring the same miRNA response element (MRE) can modulate each other’s expression levels by competitively binding to shared miRNAs that block target mRNAs [17]. In cancer, it is appreciated that lncRNAs can contribute to tumor progression and drug resistance [18–20]. For example, the lncRNA PCA3 has been identified as a novel diagnostic marker of prostate cancer progression [21] and simultaneously its lncRNA-based therapies are also formulated [22]. However, it largely remains unknown with regards to a variety of novel lncRNAs in the pathological regulation of GBC.

In the current study, based on our previous microarray data analysis, we identified a new lncRNA highly expressed in GBC, termed lncRNA highly expressed in gallbladder cancer (lncRNA-HGBC) as a key regulator of GBC growth and metastasis. We demonstrate that lncRNA-HGBC specifically binds to RNA binding protein HuR that in turn stabilizes lncRNA-HGBC. In addition, lncRNA-HGBC regulates miR-502-3p/SET/AKT axis by directly binding to and sequestering miR-502-3p that inhibits SET gene expression, thereby leading to the activation of AKT downstream pathway. To this end, our findings establishing HuR/lncRNA-HGBC/miR-502-3p/SET/AKT regulatory axis may offer novel targets for GBC therapy.

## Material and methods

### Patients and clinical specimens

Human GBC samples and adjacent benign gallbladder tissues were obtained from patients who underwent cholecystectomy without receiving preoperative chemotherapy, radiotherapy, or androgen therapy at the Department of General Surgery, Xinhua Hospital, School of Medicine, Shanghai Jiao Tong University between 2008 and 2013. Written informed consent was obtained from all participants. This study was approved by the ethics committee of Xinhua hospital. For quantitative real-time PCR analysis (qRT-PCR), tumor tissues as well as the adjacent non-tumor tissues were snap-frozen in liquid nitrogen and stored at − 80 °C. For immunohistochemical staining, each tissue sample was fixed in 4% formalin immediately after removal and embedded in paraffin.

### 5′ and 3′ rapid amplification of cDNA ends (RACE) analysis

Total RNA was isolated using TRIzol Plus RNA Purification Kit (Invitrogen), according to the manufacturer’s instructions. 5′ RACE and 3′ RACE were performed using GeneRacer™ Kit (Invitrogen) according to the manufacturer’s instructions. The following gene-specific primers (GSP) are used for PCR: 5′-CCCCTGGAGGAGGTGGAGCTTACAGAA-3′ (5′ RACE GSP1), 5′-GTGGCTCATGCCTGTAATCCCAACACTTT-3′ (5′ RACE GSP2), 5′-CCAGGTTAGTTCCTTCTGTAAGCTCCACCTC-3′ (3′ RACE GSP1), 5′-CACCCGCTAATTGGCTCCCTCAGATC-3′ (3′ RACE GSP2).

### Northern blot analysis

Northern blot was performed to characterize the full length of lncRNA-HGBC as previously described [23] with minor modifications. Briefly, 30 μg of indicated RNA was separated by formaldehyde gel electrophoresis and then transferred to a Biodyne Nylon membrane (Pall, NY, USA) and fixed by UV crosslinking. After prehybridization in Ultrahyb buffer (Ambion, Grand Island, NY) at 62 °C for 60 min, the membrane was hybridized in Ultrahyb buffer with digoxin-labeled probes for lncRNA-HGBC or β-actin at 62 °C overnight. The membrane was then washed 2 times with 2 × SSC at 62 °C for 5 min and incubated with anti-DIG-biotin antibody (BOSTER Biological Technology, BM0040) for 2 h at RT. Then the membrane was washed 2 times with 2 × SSC at 62 °C for 5 min and incubated with HRP-conjugated Streptavidin (BOSTER Biological Technology, BA1088) for 30 min at RT. After washing for another 2 times with 2 × SSC, the expression of lncRNA-HGBC was detected. The probe sequences were listed in Additional file 1: Table S1.

### Fluorescence in situ hybridization

Fluorescence in situ hybridization (FISH) was performed as previously described [23]. Briefly, NOZ and SGC-996 cells grown on the slides were washed with PBS and fixed in 4% paraformaldehyde. After protease reagent treatment, the slides were incubated with prehybridization buffer at 40 °C for 4 h, and then hybridized with digoxin-labeled probe at 40 °C overnight. After washing and blocking, the slides were incubated with biotin conjugated anti-digoxin antibody. The slides were then incubated with SABC-FITC at 37 °C for 30 min after washing. The images were captured using a confocal microscope. The probe sequence was listed in Additional file 1: Table S1.

### In vitro translation

Transcription and translation assays were performed using TNT® T7 Quick Coupled Transcription/Translation Systems and Transcend™ Non-Radioactive Translation Detection Systems (Promega) according to the manufacturer’s instructions. Briefly, 1 μg pBluescript II SK-HGBC plasmids or 1 μg T7 Luciferase Control DNA(positive control) were assembled appropriately in a 0.5 ml micro-centrifuge tube in the presence of Biotin-Lysyl-tRNA. Then the mixture was incubated at 30 °C for 90 min. The translation products were subjected to SDS-PAGE using 4–20% gradient gel and transferred to PVDF membrane. After blocking, the membrane was incubated with an anti-Streptavidin-HRP antibody at 4 °C overnight. The signal was detected using ECL reagent.

### Dual-luciferase reporter assay

Based on bioinformatic prediction [24], 6 miRNAs were selected as candidate targets of lncRNA-HGBC. pmirGLO Dual-Luciferase miR Target Expression Vector (Promega) was used to assess the direct binding of potential miRNAs to lncRNA-HGBC. The wild-type reporter construct pmirGLO-HGBC or the mutant reporter construct pmirGLO-HGBC-mut(miR-502-3p) was cotransfected with miR-502-3p mimic or miR-Control in 293 T cells. After transfection for 24 h, Firefly luciferase levels were measured using a Dual-Luciferase Reporter Assay System (Promega, Wisconsin) and normalized to Renilla luciferase activity. Each experiment was repeated at least three times.

### RNA pull-down and mass spectrometry

Biotin-labelled lncRNA-HGBC was first transcribed in vitro from pBluescript II SK-lncRNA-HGBC using Biotin RNA Labeling Mix (Roche, Germany) by SP6(for anti-sense)/T7(for sense) RNA polymerase (Roche) according to the manufacturer’s instructions. The RNA products were treated with RNase-free DNase I (Roche) and purified with an RNeasy Mini Kit (Qiagen, Valencia, CA). Four microgram biotinylated RNAs were denatured for 5 min at 65 °C in PA buffer (10 mM Tris HCl pH 7.5, 10 mM MgCl2, 100 mM NH4Cl) and slowly cooled down to room temperature. Then, the folded RNA was incubated with streptavidin Dynabeads (Invitrogen) for 1 h at 4 °C in the presence of 2 U/ml RNasin (Promega). After washing 4 × 5 min with wash buffer (10 mM HEPES pH 7.0, 400 mM NaCl, 1 mM DTT, 1% Triton X-100, protease inhibitor cocktail (Roche), 2 mM RVC), the protein lysate from 1 × 10^7^ NOZ cells was pre-cleared by streptavidin Dynabeads (Invitrogen) and incubated with the folded RNA-beads complex for 3.5 h at 4 °C in the presence of 20 μg/ml yeast tRNA. After extensive washing, beads were boiled 40 μl of 1× SDS loading buffer for 10 min at 100 °C. The lncRNA-interacting proteins were further separated by sodium dodecyl sulphate-polyacrylamide gel electrophoresis and the gel was silver stained. Then, lncRNA-HGBC specific bands were subjected to mass spectrometry and retrieved in human proteomic library.

### Isolation of cytoplasmic and nuclear RNA

Cytoplasmic and nuclear RNAs of NOZ cells were extracted and purified using PARIS™ Kit (Invitrogen) according to the manufacturer’s instructions.

### RNA immunoprecipitation (RIP) assay

RIP was performed using a Magna RIP RNA-Binding Protein Immunoprecipitation kit (Millipore, Bedford, MA) according to the manufacturer’s instructions. Briefly, 2 × 10^7^ NOZ cell lysates were incubated with magnetic beads conjugated with negative control normal mouse IgG or human anti-Ago2 antibody (Millipore). The immunoprecipitated RNAs were then extracted and detected by qRT-PCR to confirm the enrichment of binding targets and the products were then subjected to agrose gel electrophoresis. The primers used for detecting lncRNA-HGBC or miR-502-3p were listed in Additional file 1: Table S2.

### MS2-RIP

We co-transfected pcDNA3.1-MS2, pcDNA3.1-MS2-HGBC, pcDNA3.1-MS2-HGBC-MUT(502-3p) along with pMS2-GFP(Addgene) into NOZ cells using Viafect reagent. After 48 h, cells were collected and lysed to perform RNA immunoprecipitation (RIP) experiments using a GFP antibody (Roche) and the Magna RIP™ RNA-Binding Protein Immunoprecipitation Kit (Millipore, Bedford, MA) according to the manufacturer’s instructions. Finally, purified RNAs were isolated and determined by real-time PCR to confirm the presence of binding targets. The primers used for detecting miR-502-3p or miR-122 were provided in Additional file 1: Table S2.

### Statistical analysis

All statistical analyses were performed using SPSS 19.0 software. Each experiment was performed in triplicate, and the data were shown as the mean ± SD, unless otherwise stated. Kaplan-Meier analysis with log-rank test was used for survival analysis. Student’s t-test was used to compare the mean values. Pearson chi-square test was used to analyze the association between lncRNA-HGBC expression and clinicopathologic parameters. *P* value< 0.05 was considered to be statistically significant.

### Supplemental materials and methods

Supplemental Materials and Methods were provided as Additional file 2 and Additional file 1: Table S1-S3.

## Results

### LncRNA-HGBC was identified and its high expression was correlated with poor prognosis of GBC

**Fig. 1:**
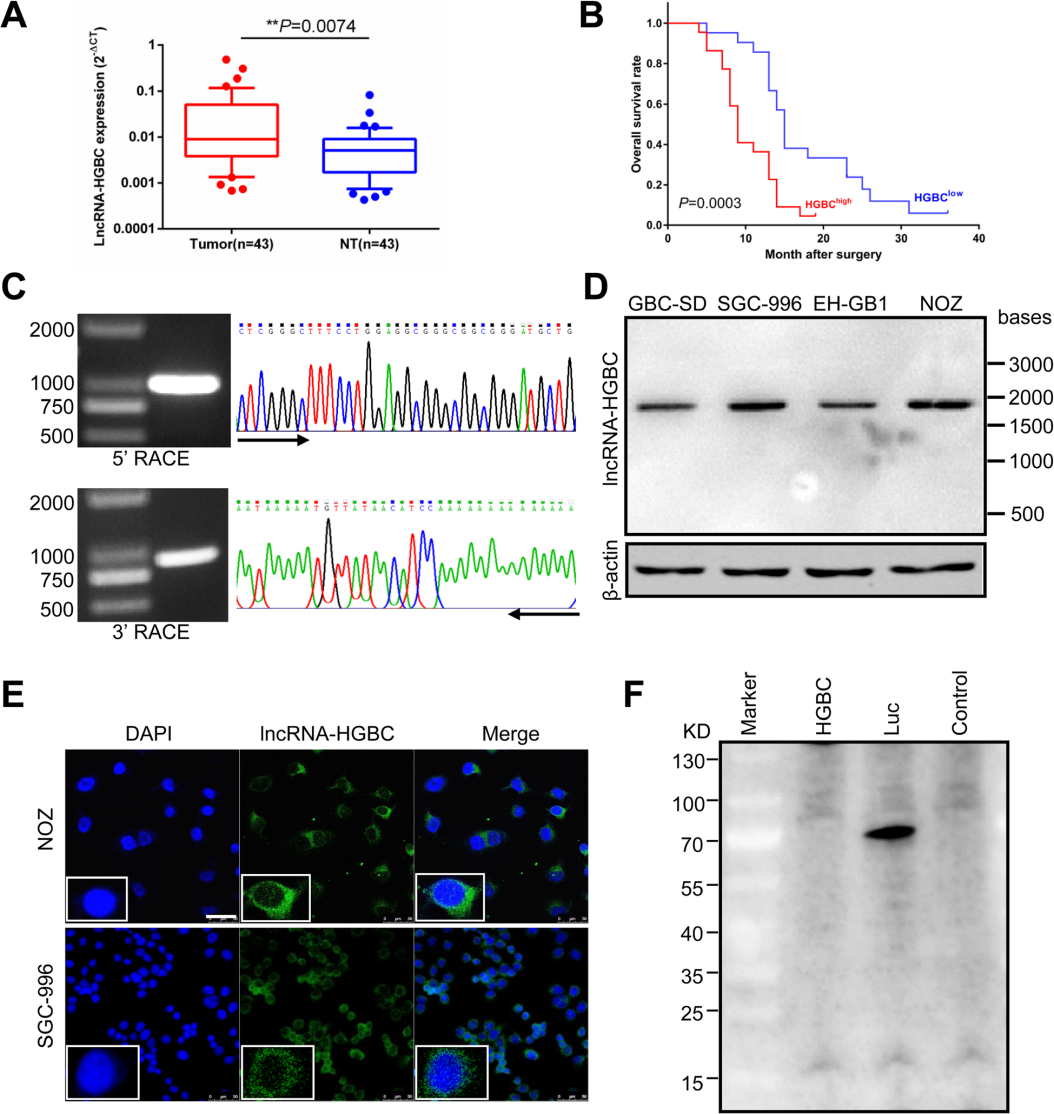
Identification of a novel lncRNA-HGBC whose expression levels were associated with the progression of GBC. **a** Box and whisker plots of the relative expression of lncRNA-HGBC in 43 paired human GBC tissues and non-tumour tissues (NT). **b** Kaplan-Meier survival curves of 43 gallbladder cancer patients with the high- and low-lncRNA-HGBC expression. The median expression level of lncRNA-HGBC was used as the cutoff. **c** 5′ and 3′ rapid amplification of cDNA ends (RACE) assays in NOZ cells to detect the whole sequence of lncRNA-HGBC. Left; a gel electrophoresis image of PCR products from the 5′-RACE and 3′-RACE assays. Right; sequencing of PCR products indicated the boundary between the universal anchor primer and lncRNA-HGBC sequences. **d** Northern blot analysis to confirm the length and expression of the lncRNA- HGBC in GBC tissues and cells. β-Actin was used as a loading control. **e** Fluorescence in Situ Hybridization (FISH) assay (green) used to examine the expression and location of lncRNA-HGBC in SGC-996 and NOZ cells. Scale bars, 50 μm. **f** In vitro translation assay using luciferase (Luc) as a positive control and lncRNA-HGBC templates as described in the Methods

We previously performed a microarray analysis to compare both lncRNA and mRNA differential expression profile between GBC and adjacent benign tissues [23] (Gene Expression Omnibus(GEO) accession number: GSE76633). In the top candidate lists of differentially expressed lncRNAs, we noted that a lncRNA labeled as NR_027005, which has a high rate of coexpressed protein-coding RNAs, was increased by 16.6 times in GBC tissue relative to non-tumor tissue (Additional file 3: Figure S1A). Therefore, we were particularly focused on this uncharacterized lncRNA and named lncRNA-HGBC (lncRNA Highly expressed in GBC). Coexpression network analysis showed that lncRNA-HGBC was biologically associated with other 17 lncRNAs and 42 protein-coding genes (Additional file 1: Table S4 and Additional file 3: Figure S1B). To further validate the increased level of lncRNA-HGBC in GBC, we examined lncRNA-HGBC expression in another set of 43 cases containing both cancer and adjacent non-tumor tissues, and found that lncRNA-HGBC level was significantly higher in GBC tissues than in benign tissues (*P* = 0.0074; Fig. 1a). Next, to determine if lncRNA-HGBC expression level is related to GBC progression, we analyzed the association between lncRNA-HGBC levels and clinicopathological characteristics in those GBC patients. Using the median expression level of lncRNA-HGBC as a cutoff, 43 GBC patients were stratified into two groups with low and high lncRNA-HGBC expression. As shown in Table 1, statistical analyses showed that high lncRNA-HGBC level was positively correlated with TNM stage (*P* = 0.0096) and lymph node metastasis (*P* = 0.0191). Accordingly, Kaplan-Meier and log-rank tests indicated that high lncRNA-HGBC expression levels were significantly correlated with reduced overall survival (OS) (*P* < 0.001, Fig. 1b), implicating an active role in cancer metastasis.

**Table 1:** Association between lncRNA-HGBC expression and clinicopathologic characteristics of GBC patients in the study cohort

Characteristics	Cases	LncRNA-HGBC expression		χ^2^ value	*P* value
		Low (%)	High (%)		
Sex				0.297	0.585
*Male*	14	6 (28.6%)	8 (36.4%)		
*Female*	29	15 (71.4%)	14 (63.6%)		
Age (years)				0.009	0.924
*< 60*	12	6 (28.6%)	6 (27.3%)		
*≥ 60*	31	15 (71.4%)	16 (72.7%)		
Histology differentiation				0.007	0.933
*Well or moderate*	33	16 (76.2%)	17 (77.3%)		
*Poor*	10	5 (23.8%)	5 (22.7%)		
**TNM stage (AJCC)**				**6.702**	**0.0096**
*0-II*	20	14 (66.7%)	6 (27.3%)		
*III-IV*	23	7 (33.3%)	16 (72.7%)		
**Lymph node Metastasis**				**5.495**	**0.0191**
*Present*	18	5 (23.8%)	13 (59.1%)		
*Absent*	25	16 (76.2%)	9 (40.9%)		

*AJCC* American Joint Committee on Cancer, Bold type indicates statistical significance

The full length of lncRNA-HGBC was successfully obtained by rapid amplification of the 5′ and 3′ cDNA ends (RACE) assays (Fig. 1c and Additional file 3: Figure S1C). We confirmed the full-length sequence of lncRNA-HGBC by running PCR with 3 pairs of fragmented primers (Additional file 3: Figure S1D). Consistent with the RACE data, Northern blot validated that RNA full length of lncRNA-HGBC was ~ 2 kb in length in GBC cell lines (Fig. 1d). To further determine the subcellular localization of lncRNA-HGBC, we separated the nuclear and cytoplasm fraction of NOZ cells and performed qRT-PCR. The results suggested that this lncRNA was mainly located in the cytoplasm (Additional file 3: Figure S1E). Fluorescence in situ hybridization (FISH) analysis indeed revealed this cytoplasm distribution (Fig. 1e). In order for this gene product representing non-protein coding RNA, we employed the sequence analysis program by ORF Finder from the National Center for Biotechnology Information and the result showed that it failed to predict a protein of more than 90 amino acids (Additional file 3: Figure S1F). The coding probability of lncRNA-HGBC was as low as 0.029 if any, as calculated by Coding-Potential Assessment Tool (CPAT) [25] (Additional file 3: Figure S1G). In addition, codon substitution frequency (CSF) analysis indicated that lncRNA-HGBC did not have protein-coding potential (Additional file 3: Figure S1H). Furthermore, the full-length lncRNA-HGBC was lack of the ability to express any protein using in vitro translation assay (Fig. 1f), underscoring that LncRNA-HGBC is a non-protein coding RNA.

### LncRNA-HGBC promotes GBC cell proliferation in vitro and in vivo

**Fig. 2:**
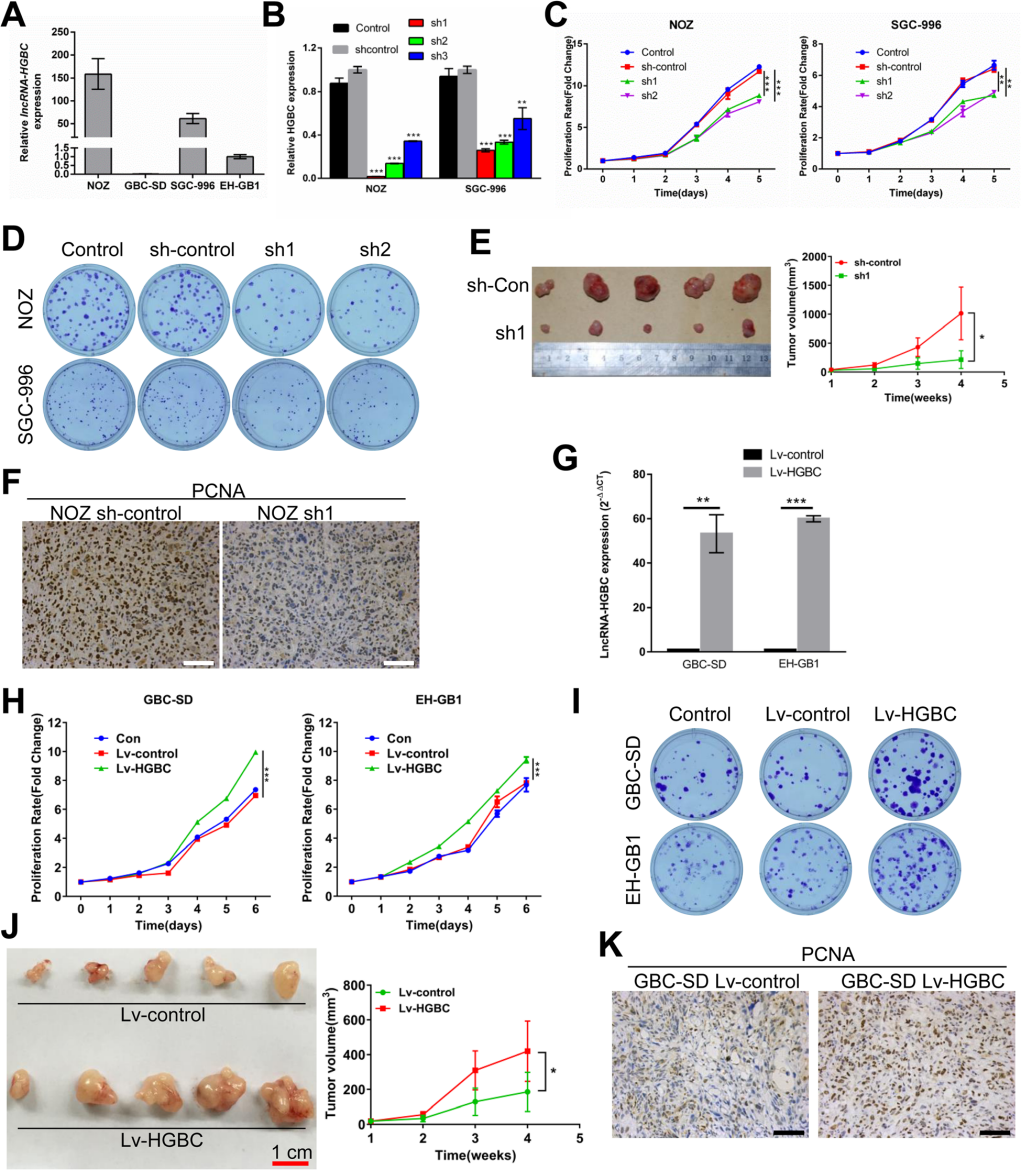
LncRNA-HGBC promotes GBC cell proliferation and tumor growth. **a** The relative lncRNA-HGBC expression was measured using qRT-PCR in the indicated GBC cell lines. **b** LncRNA-HGBC expression level was detected in NOZ and SGC-996 cells by qRT–PCR after viral infection. *P < 0.05. **c** Cell proliferation assays for NOZ and SGC-996 cells expressing shRNA sh1, sh2 or the negative control (sh-control) were determined using CCK-8 assays. **d** Typical photographs of colony formation assays of lncRNA-HGBC knockdown or control control NOZ (top) and SGC-996 cells (bottom) were shown. **e** Effects of lncRNA-HGBC knockdown in NOZ cells on tumor growth in vivo. Left, representative images of tumors formed in nude mice (*n* = 5). Right, tumor volumes were measured once a week and tumor growth curves are summarized in the line chart (*P < 0.05). **f** PCNA expression was examined in sections of NOZ xenografts by immunohistochemistry. Scale bars, 100 μm. **g** lncRNA-HGBC expression level was determined in GBC-SD and EH-GB1 cells by qRT–PCR. **h** Cell proliferation assays for GBC-SD and EH-GB1 cells expressing lncRNA-HGBC or the control. **i** Colony formation assays of lncRNA-HGBC-overexpressing or control GBC-SD (top) and EH-GB1 cells (bottom). **j** Images of tumor formation in nude mice (*n* = 5) injected subcutaneously with GBC-SD cells overexpressing lncRNA-HGBC (bottom) or the control (top). Tumor volumes were measured once a week and tumor growth curves are summarized in the line chart.(*P < 0.05). **k** PCNA expression in sections of GBC xenografts was determined by immunohistochemistry. Scale bars, 100 μm

Next, we sought to determine effects of lncRNA-HGBC on GBC cell proliferation and tumorigenesis. First, we measured the expression of lncRNA-HGBC in four GBC cell lines and found that NOZ and SGC-996 cells showed higher levels of lncRNA-HGBC than GBC-SD and EH-GB1 (Fig. 2a). Then, we stably silenced lncRNA-HGBC via gene shRNA in NOZ and SGC-996 cell lines (Fig. 2b and Additional file 3: Figure S2A). Knockdown of lncRNA-HGBC in either one of two shRNAs led to significantly decreased cell proliferation over a 5-day culture (Fig. 2c). Consistent with the proliferation data, silencing lncRNA-HGBC by shRNA1 and shRNA2 also notably decreased the colony formation ability of both cell lines to form colony (Fig. 2d and Additional file 3: Figure S2B). Furthermore, to determine the effects of lncRNA-HGBC on GBC growth in vivo, lncRNA-HGBC-knockdown or control NOZ cells were injected subcutaneously into nude mice. The results showed that tumor volume and tumor weight in mice injected with lncRNA-HGBC-knockdown NOZ cells were significantly decreased to approximately 30% of those developed in control mice (Fig. 2e and Additional file 3: Figure S2C). Agreed with these data, cell proliferation using proliferating cell nuclear antigen (PCNA) assay unveiled a decreased level of cell proliferation in lncRNA-HGBC-depleted tumors compared with the control group (Fig. 2f).

To further confirm the effect of lncRNA-HGBC on GBC tumorigenesis, we employed a complementary approach by developing cells to stably overexpress lncRNA-HGBC in GBC-SD and EH-GB1 cells that express low levels of endogenous lncRNA-HGBC (Fig. 2g and Additional file 3: Figure S2D). As expected, LncRNA-HGBC-overexpressing cells (Lv-HGBC) showed increased cell proliferation and cell colonies compared with empty vector-transfected cells (Lv-control) (Fig. 2h, i and Additional file 3: Figure S2E). In addition, tumor volume and weight were 2.5-fold greater in the lncRNA-HGBC-overexpressing group than those in the controls (Fig. 2j and Additional file 3: Figure S2F). IHC staining showed that PCNA was upregulated in lncRNA-HGBC-overexpressing xenograft tumor tissues (Fig. 2k). Collectively, our data demonstrate that lncRNA-HGBC acts as a tumor-promoting factor to enhance tumorigenesis of GBC cells.

### LncRNA-HGBC drives GBC cells to undergo EMT and cancer metastasis

**Fig. 3:**
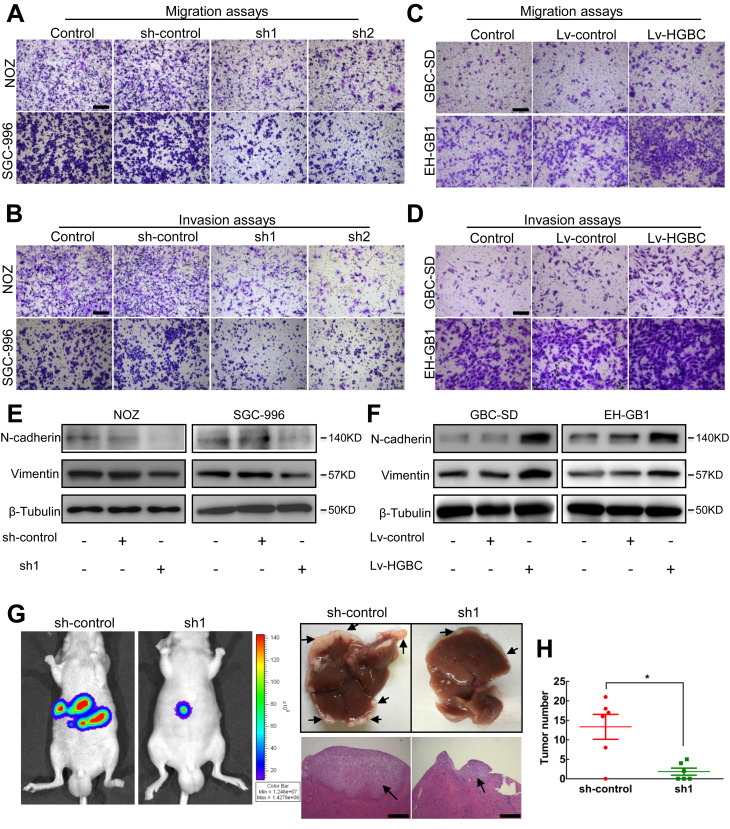
LncRNA-HGBC reinforces the invasive capacity of GBC cells. **a, b** Transwell assays (**a**) and Invasion assays (**b**) were used in NOZ and SGC-996 cells. Scale bars, 200 μm. **c**, **d** Transwell assays (**c**) and Invasion assays (**d**) were used in lncRNA-HGBC-overexpressing GBC-SD and EH-GB1 cells. Scale bars, 200 μm. **e, f** The protein levels of N-cadherin and Vimentin in control and lncRNA-HGBC-knockdown NOZ (left) or SGC-996 (right) cells, and in control and lncRNA-HGBC-overexpressing GBC-SD (left) or EH-GB1 (right) cells. **g** Representative images of luciferase signals in mice at the 6 weeks after intrasplenic injection with NOZ cell clones (left). Representative livers were shown and the isolated liver tissues sections were stained by hematoxylin and eosin (righ). Arrows indicate the metastasis nodules. Scale bars, 500 μm. **h** The average number of liver metastases in the intrasplenic injection model. Data are presented as mean ± SD of three independent experiments. *P < 0.05, **P < 0.01, ***P < 0.001

Given that the lncRNA-HGBC RNA level was positively related to the existence of lymph node metastasis, we reasonably postulate that lncRNA-HGBC augments GBC cell invasive behavior. To test the hypothesis, we exploited cell transwell migration and matrigel invasion assays, and found that knocking down endogenous lncRNA-HGBC by specific shRNAs dramatically reduced the cell migration and invasion by around 30–40% of controls in NOZ and SGC-996 cells (Fig. 3a, b and Additional file 3: Figure S3A, B). Conversely, overexpression of lncRNA-HGBC in GBC-SD and EH-GB1 cells increased the migration and invasion by 20–50% (Fig. 3c, d and Additional file 3: Figure S3C, D). It is well established that increased metastatic capability of GBC is intimately associated with tumor cell phenotypic transformation, an event termed epithelial-mesenchymal transition (EMT) [26]. To evaluate the possibility of EMT involved in the invasiveness of GBC, we monitored EMT-specific markers such as vimentin and N-cadherin. lncRNA-HGBC knockdown in NOZ and SGC-996 cells decreased expression of vimentin and N-cadherin (Fig. 3e). On the contrary, lncRNA-HGBC overexpression in GBC-SD and EH-GB1 led to increased both levels (Fig. 3f). To confirm these findings in vitro and visualize the likelihood of acquired tumor metastasis in vivo, we established a liver metastasis tumour model in nude mice by injecting NOZ cells to the spleen. Five out of the six mice (5/6) in the control group showed increased luciferase signals and intrahepatic metastatic nodules in their livers after 6 week-transplantation, whereas only three of the six mice (3/6) injected with lncRNA-HGBC-shRNA NOZ cells developed liver nodules. Strikingly, metastatic foci seen in the liver in lncRNA-HGBC-shRNA tumor mice was as low as 10% of control metastatic tumors (Fig. 3g, h). Taken together, these data strongly suggest that lncRNA-HGBC commits GBC cells to undergoing EMT and promoting tumor metastasis.

### HuR physically interacts with and stabilizes lncRNA-HGBC

**Fig. 4:**
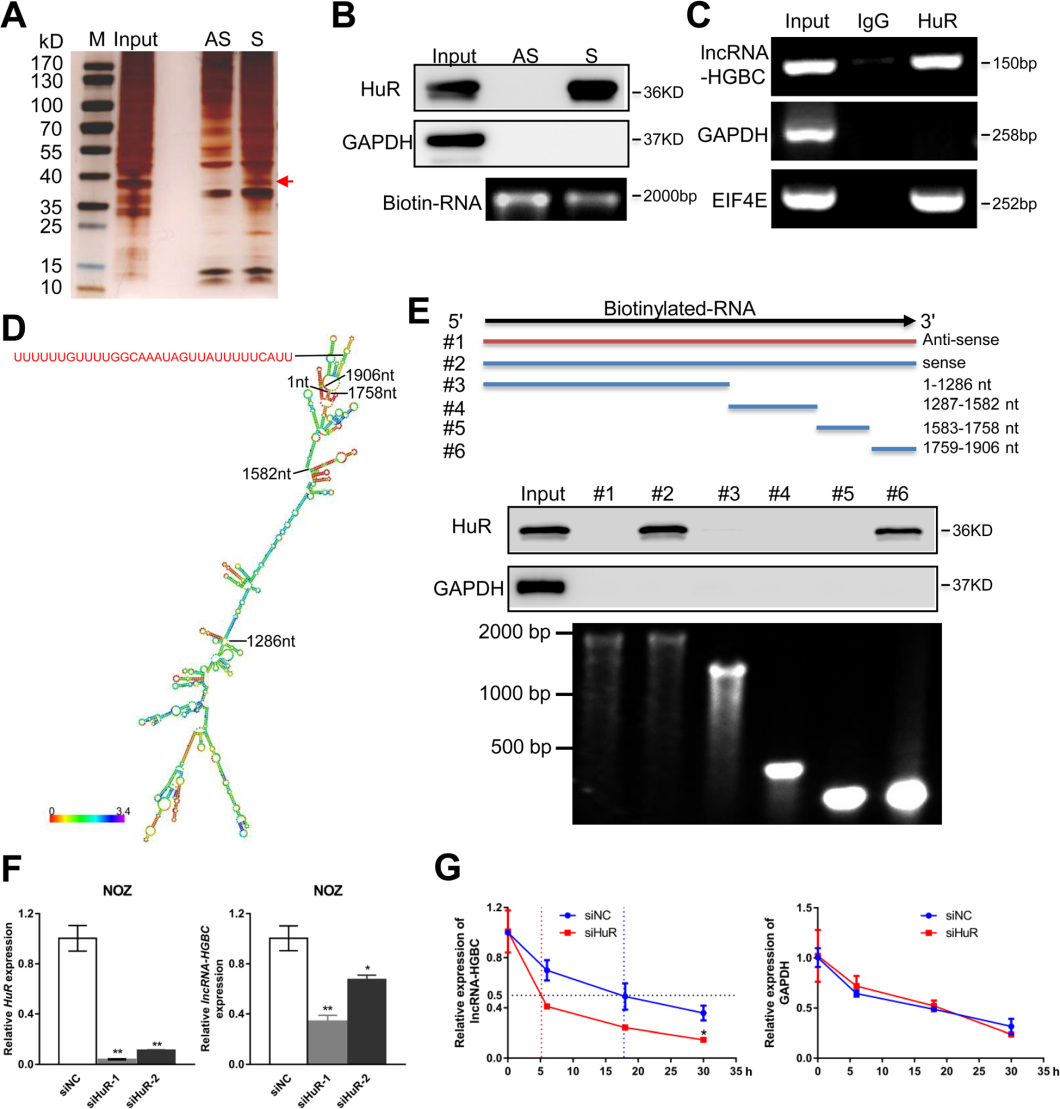
HuR physically interacts with and stabilizes lncRNA-HGBC. **a** RNA pull down assay by lncRNA-HGBC and its antisense RNA followed by silver staining of protein extract from NOZ cells. A band indicated by an arrow was excised for mass spectrometry analysis. S: sense strand of lncRNA-HGBC, AS: anti-sense strand of lncRNA-HGBC. **b** Western blot analysis of the specific association of HuR and lncRNA-HGBC. GAPDH was used as the negative control. S: sense strand of lncRNA-HGBC, AS: anti-sense strand of lncRNA-HGBC. **c** RIP experiments were performed using an antibody against HuR on NOZ cell extracts followed qRT-PCR. GAPDH and EIF4E were served as the negative and positive control, respectively. **d** The predicted secondary structure of lncRNA-HGBC. **e** Immunoblotting of HuR in pull-down samples by full-length biotinylated-lncRNA-HGBC (#2), antisense lncRNA-HGBC (#1) or truncated biotinylated-lncRNA-HGBC fragments (#3: 1–1286 nt; #4: 1287–1582 nt; #5: 1583–1758 nt; #6: 1759–1906 nt), with GAPDH as the negative control. The bottom image showed each transcribed RNA. **f** qRT-PCR analysis of HuR and lncRNA-HGBC levels in HuR-depleted NOZ cells. **g** NOZ cells transfected HuR siRNA or siNC were treated with α-amanitin (50 mM) to block new RNA systhesis and the levels of lncRNA-HGBC and β-actin were assessed by qRT-PCR analysis and normalized to 18S rRNA (a product of RNA polymerase I that is unaffected by α-amanitin). All values at time 0 h were normalized to 1. ***P < 0.001, **P < 0.01, *P < 0.05

Growing evidence has pointed to the notion that many lncRNAs can function to regulate some target gene expression through direct interaction with proteins [27–29]. To explore the potential binding proteins for lncRNA-HGBC in GBC cells, we performed a biotin-labeled RNA pull-down assay followed by silver staining (Fig. 4a). A protein band specifically presented in lncRNA-HGBC was located at approximately 37 kD and then was subjected to sequence analysis via mass spectrometry. With great interest in the analyzed data (Additional file 1: Table S5), we paid particular attention to an RNA-binding candidate HuR that was confidence score > 100 and molecular weight (MW) 30–40 KD. Western blot assay confirmed that HuR was a specific binding protein for lncRNA-HGBC (Fig. 4b). An RNA immunoprecipitation (RIP) assay was further utilized to validate the specific interaction between lncRNA-HGBC and HuR, as compared with the positive control EIF4E mRNA [30] that is enriched in HuR binding (Fig. 4c). Next, to interrogate which specific region within lncRNA-HGBC contributes to HuR binding, we constructed four different deletion fragments of lncRNA-HGBC based on the secondary structure of lncRNA-HGBC that was predicted from RNA fold Web server (http://rna.tbi.univie.ac.at/cgi-bin/RNAfold.cgi) (Fig. 4d). Subsequently, RNA pulldown assay followed by WB showed HuR specific binding sequence was located within 1759–1906 nt-long region that harbors HuR-binding motif (UUUUUUGUUUUGGCAAAUAGUUAUUUUUCAUU) (Fig. 4e), indicating that 1759–1906 nt renders lncRNA-HGBC able to bind to HuR.

To investigate possible effects of lncRNA-HGBC on HuR expression, we measured protein expression of HuR in lncRNA-HGBC-overexpressing GBC-SD and lncRNA-HGBC-knockdown NOZ cells. The results showed that lncRNA-HGBC did not have ability to change HuR expression (Additional file 3: Figure S4). Given the rigorous activity of HuR in stabilizing mRNAs and lncRNAs [31, 32], we examined whether HuR affects the stability of lncRNA-HGBC in GBC cells. As shown in Fig. 4f, the expression of lncRNA-HGBC in NOZ cells was reduced by 65.8 and 32.5% in two independent HuR-knockdown cells, respectively. To further investigate if this reduction was due to increased lncRNA-HGBC decay, we incubated NOZ cells with α-amanitin to block de novo RNA transcription and then measured the expression of lncRNA-HGBC over a 30 h period. The depletion of HuR decreased the half-life of lncRNA-HGBC level from 18 h to 5 h (Fig. 4g), indicating that HuR contributed to stabilizing lncRNA-HGBC. Collectively, these data strongly suggest that HuR interacts with and stabilizes lncRNA-HGBC.

### LncRNA-HGBC functions as a competing endogenous RNA by directly binding to and inhibiting miR-502-3p

**Fig. 5:**
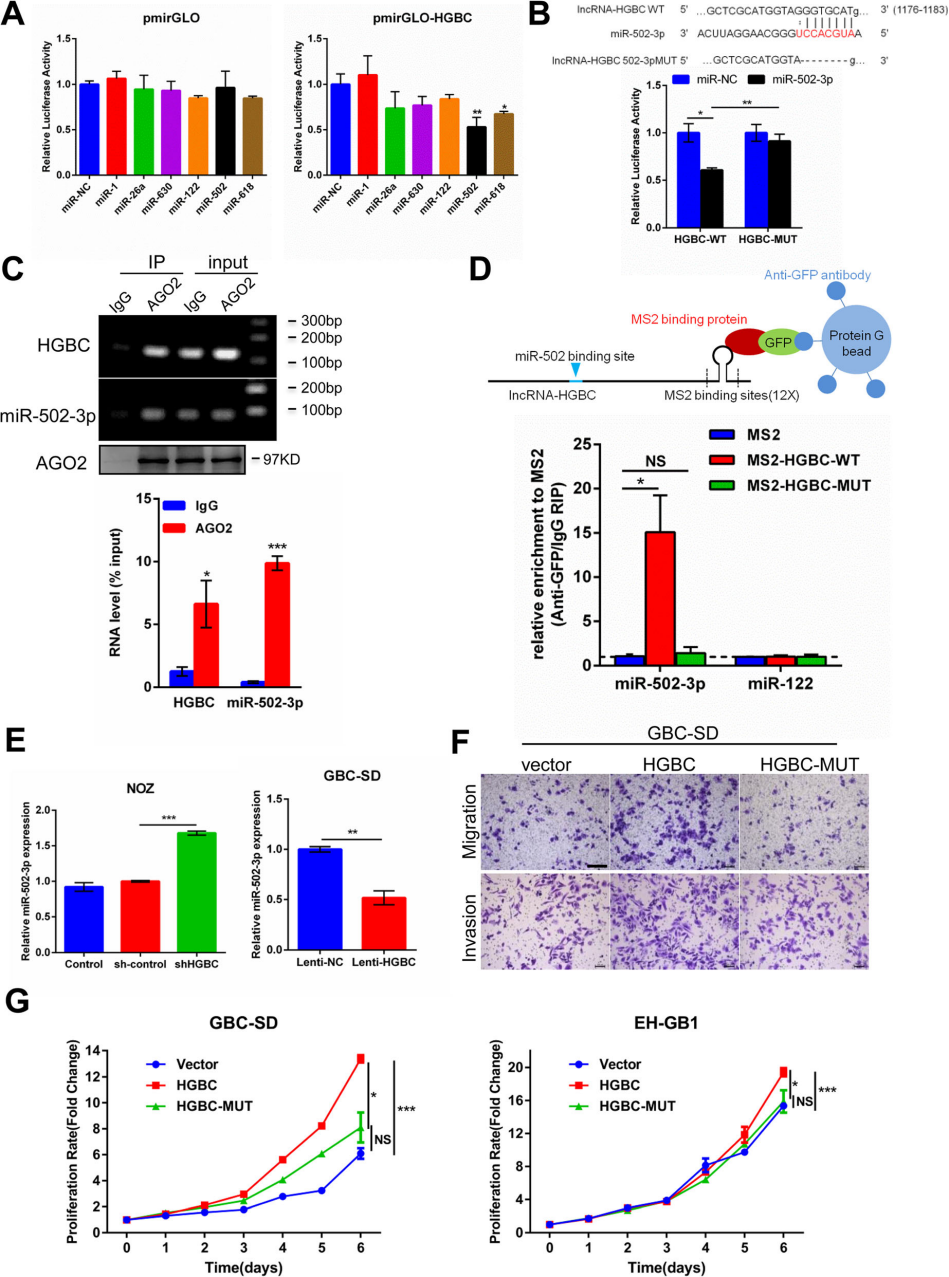
LncRNA-HGBC functions as a competing endogenous RNA by directly binding to miR-502-3p. **a** Luciferase activity in 293 T cells expressing lncRNA-HGBC binding miRNAs and empty luciferase reporter (left) or lncRNA-HGBC luciferase reporter (right). **b** Wild type and mutant lncRNA-HGBC sequences were cloned into pmirGLO vectors and co-transfected with miR-502-3p into 293 T cells followed by dual luciferase assay. **c** Anti-Ago2 RIP was used to pulldown endogenous RNAs associated with Ago2; IgG was served as the control. Ago2 in proteins from Ago2-RIP assay were measured by western blot. The levels of lncRNA-HGBC and miR-502-3p were measured by qRT–PCR and the data were presented as fold enrichment in Ago2 relative to input (*P < 0.05, ***P < 0.001). **d** MS2-RIP in NOZ cells followed by qRT-PCR to detect endogenous association between miR-502-3p and lncRNA-HGBC. miR-122 was a negative control. A schematic outline of the MS2-RIP strategy was shown (top). **e** qRT-PCR analysis of miR-502-3p expression in NOZ cells after knockdown of lncRNA-HGBC. **f** Transwell migration (top) and invasion (bottom) assays of GBC-SD cells that were transfected with the indicated plasmids. Scale bars, 200 μm. **g** After transfection of plasmids containing vector control, lncRNA-HGBC or lncRNA-HGBC-MUT, the proliferation of GBC-SD and EH-GB1 cells was measured using CCK-8 assays. *P < 0.05, ***P < 0.001

Emerging lines of evidence have reported that cytoplasm lncRNAs can function as competing endogenous RNAs (ceRNAs) by binding to and sequestering specific miRNAs that block target gene expression [19, 20]. Given that lncRNA-HGBC is mainly located in the cytoplasm, we hypothesized that lncRNA-HGBC may function as miRNA sponge to restore gene expression targeted by miRNA in GBC progression. In a set of miRNAs that are putatively bound to lncRNA-HGBC in the Segal Lab program (Eran Segal; http://132.77.150.113/pubs/mir07/mir07_prediction.html) [24] (Additional file 1: Table S6), we were particularly interested in six tumor suppressor-associated miRNAs including miR-1, miR-26a, miR-630, miR-122, miR-502-3p and miR-618. To obtain the bona fide lncRNA-miRNA interaction, we subcloned full-length lncRNA-HGBC into the pmirGLO dual luciferase reporter vector. Dual luciferase assay showed that miR-502-3p and miR-618 could suppress the luciferase activity of lncRNA-HGBC, but not other four miRNAs (Fig. 5a), indicating a possible interaction between lncRNA-HGBC and miR-502-3p or miR-618. We unexpectedly found miR-502-3p was indeed downregulated in GBC tissues compared with adjacent non-tumor tissues based on our previous miRNA microarray results [33] (data not shown, GEO accession number GSE90001). Therefore, we were primarily focused on the interaction between lncRNA-HGBC and miR-502-3p. Once miR-502-3p binding motif of GGTGCAT between 1176 and 1183 nt of lncRNA-HGBC was deleted as mutant HGBC-MUT, luciferase activity was not suppressed, as compared with decreased activity in the presence of wild type of miR-502-3p binding motif (Fig. 5b). A large body of mechanistic studies focusing on the interaction between miRNA and targeted mRNA have established the notion that miRNAs bind to their mRNA targets and cause translational suppression and/or RNA degradation by forming a complex with Argonaute2 (AGO2) [34]. To test this action model, RNA immunoprecipitation (RIP) experiments were employed in NOZ cell extracts using an AGO2 antibody. As shown in Fig. 5c, both lncRNA-HGBC and miR-502-3p were specifically enriched in AGO2 antibody-associated complex, but not in the control IgG, suggesting that miR-502-3p is a bona fide lncRNA-HGBC-targeting miRNA. To further identify whether lncRNA-HGBC directly binds to endogenous miR-502-3p, we performed MS2-RIP to pulldown endogenous miRNAs associated with lncRNA-HGBC (Fig. 5d, above). The results demonstrated that lncRNA-HGBC in NOZ cells was significantly associated with miR-502-3p, but not with irrelevant microRNA (miR-122). However, mutation of miR-502-3p binding site in lncRNA-HGBC abolished their association (Fig. 5d, below), supporting the direct interaction between lncRNA-HGBC and miR-502-3p.

To further clarify the regulatory relationship of gene expression between lncRNA-HGBC and miR-502-3p, we evaluated miR-502-3p levels in cells expressing divergent levels of lncRNA-HGBC. qRT-PCR results indicated that miR-502-3p was markedly upregulated by 60% after lncRNA-HGBC knockdown in NOZ cells (Fig. 5e, left). Conversely, the expression of miR-502-3p was inhibited to 50% of control after lncRNA-HGBC overexpression in EH-GB1 cells (Fig. 5e, right). However, the expression of lncRNA-HGBC was not altered after miR-502-3p overexpression or knockdown (Additional file 3: Figure S5A), suggesting that lncRNA-HGBC acts as a sponge to inhibit miR-502-3p, but did not induce degradation. According to the ceRNA feature that the expression level of lncRNAs should be comparable to their binding miRNAs, we found that the expression levels of lncRNA-HGBC and miR-502-3p in NOZ cells were approximately 382 and 450 copies per cell, respectively. To examine whether the activity of lncRNA-HGBC depends on its binding to miR-502-3p, cck-8 and Transwell assays were performed after ectopic expression of wild type lncRNA-HGBC and binding mutant HGBC-MUT. The results showed that enforced expression of lncRNA-HGBC, but not HGBC-MUT, significantly promoted cell migration, invasion and proliferation (Fig. 5f-g). As wild type lncRNA-HGBC was knocked down, the opposite effects were observed (Additional file 3: Figure S5B-S5D). Collectively, these data demonstrate that lncRNA-HGBC physically binds to miR-502-3p and may serve as a sponge to inhibit miR-502-3p.

### LncRNA-HGBC upregulates expression of SET via sequestration of miR-502-3p

miR-502-3p is appreciated to target multiple protein-coding genes including SET [35] that plays an important role in the development of various carcinomas [36, 37]. More importantly, SET was also upregulated in GBC tissues in our previous microarray data [23] (data not shown, GEO accession number: GSE76633). To decipher the regulatory mechanisms of miR-502-3p on SET, we transfected a luciferase reporter vector harboring 3′ UTR of SET into 293 T cellsand luciferase activity was then evaluated in the transfection of miR-502-3p mimics. As compared to the control vector, miR-502-3p mimics significantly reduced the luciferase activity of the SET reporter vector (Additional file 3: Figure S6A). Furthermore, after overexpression or knockdown of miR-502-3p in GBC cells (Additional file 3: Figure S6B), the expression of SET was decreased by miR-502-3p or increased by anti-miR-502-3p, respectively, at both mRNA and protein levels (Additional file 3: Figure S6C, S6D). These data indicate that SET is a direct target of miR-502-3p.

**Fig. 6:**
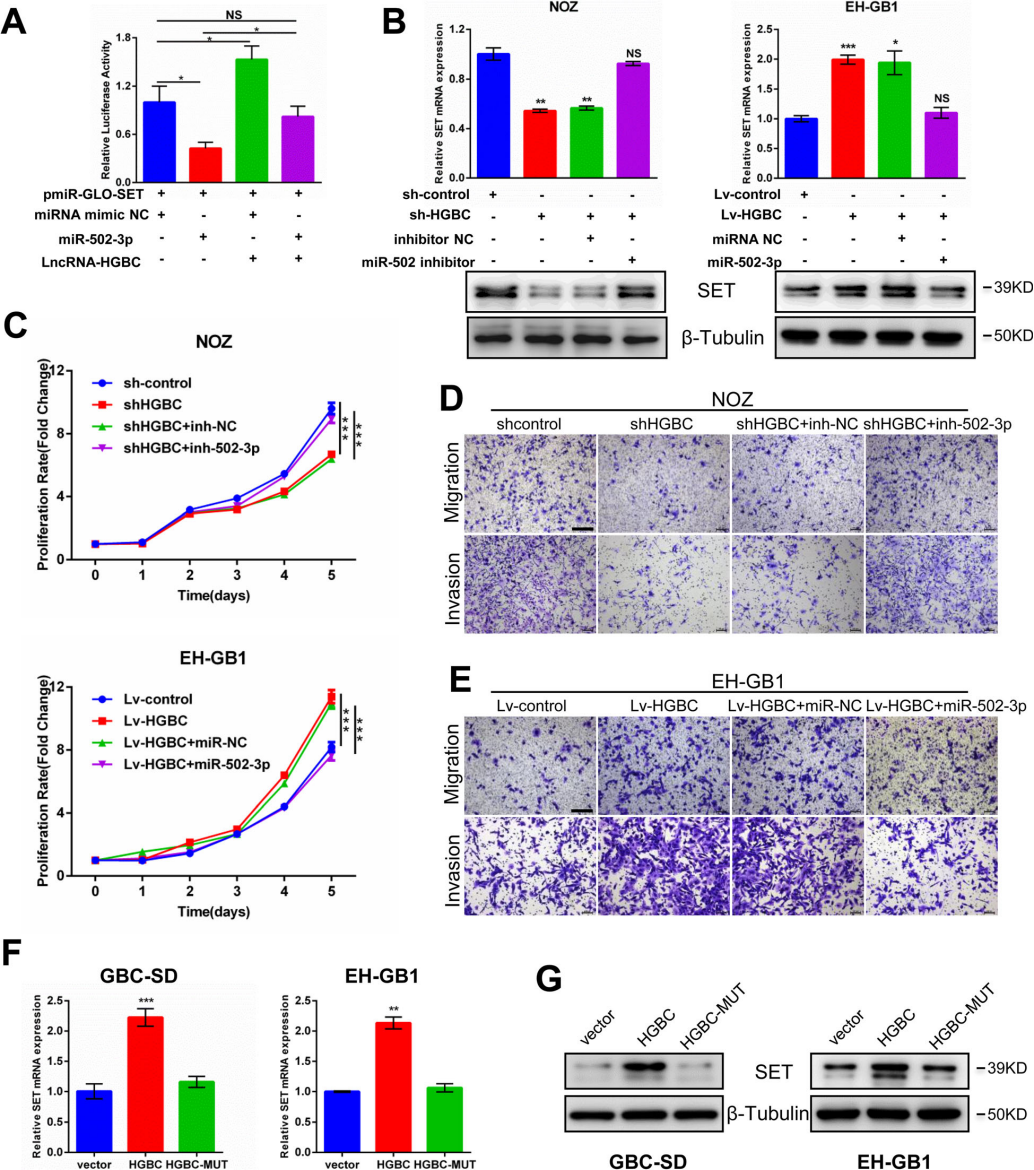
LncRNA-HGBC increases the expression of oncoprotein SET via competitively binding to miR-502-3p. **a** Dual luciferase assay was performed in NOZ cells cotransfected with miR-502-3p mimics or lncRNA-HGBC expressing plasmid and luciferase reporters containing 3’UTR of SET. **b** SET mRNA (top) and protein (bottom) levels in stably NOZ and EH-GB1 cell clones transduced with indicated DNA or RNAs. **c** CCK-8 assays in stable lncRNA-HGBC-depleted NOZ or stable lncRNA-HGBC-overexpressing EH-GB1 cells transfected with indicated miR-502-3p inhibitors or mimics, respectively. **d** Transwell migration and invasion assays were tested in NOZ cells transfected with shlncRNA-HGBC and miR-502-3p inhibitor. Scale bars, 200 μm. **e** Transwell migration and invasion assays in lncRNA-HGBC-overexpressing EH-GB1 cells were performed in co-transfection of miR-502-3p mimics. Scale bars, 200 μm. **f, g** SET mRNA (**f**) and protein (**g**) levels after transfection of the indicated plasmids into GBC-SD and EH-GB1 cells. *P < 0.05, **P < 0.01, ***P < 0.001

Since lncRNA-HGBC shares miR-502-3p binding with 3′ UTR of SET, we wondered whether lncRNA-HGBC modulates miR-502-3p-mediated inhibition of SET in GBC cells. First, we examined whether lncRNA-HGBC has the ability to inhibit miR-502-3p activity. A pmirGLO-SET luciferase reporter vector was co-transfected with lncRNA-HGBC overexpression plasmid, miRNA mimic NC and/or miR-502-3p mimic into GBC-SD cells. As shown in Fig. 6a, luciferase activity of the SET reporter vector was inhibited by overexpressing miR-502-3p but was induced by lncRNA-HGBC overexpression. Once both miR-502-3p and lncRNA-HGBC were introduced, the luciferase activity was restored to the control level, implying that lncRNA-HGBC has the ability to release SET from miR-502-3p inhibition by sequestering miR-502-3p. To provide direct evidence for regulation of SET mRNA and protein expression, we introduced lncRNA-HGBC shRNA and miR-502-3p inhibitor into NOZ cells that express high endogenous levels of lncRNA-HGBC. The results showed that lncRNA-HGBC knockdown suppressed SET expression, but the addition of miR-502-3p inhibitor increased the SET expression to the control levels (Fig. 6b, left and Additional file 3: Figure S6E, left). On the contrary, in EH-GB1 cells that express low endogenous levels of lncRNA-HGBC, overexpression of lncRNA-HGBC induced SET expression; however, the additional introduction of miR-502-3p decreased SET to the basal level (Fig. 6b, right and Additional file 3: Figure S6E, right). Accordingly, inhibition of miR-502-3p sufficiently reversed lncRNA-HGBC shRNA-inhibited cell proliferation, migration and invasion in NOZ cells (Fig. 6c, d and Additional file 3: Figure S6F). In contrast, miR-502-3p overexpression ablated increased cell growth, migration and invasion induced by lncRNA-HGBC overexpression in EH-GB1 cells (Fig. 6c, e and Additional file 3: Figure S6F). To examine whether lncRNA-HGBC-induced SET expression depends on its binding to miR-502-3p, overexpression of lncRNA-HGBC in both GBC-SD and EH-GB1 cells resulted in increases in SET expression at mRNA and protein levels, whereas miR-502-3p-binding mutation of lncRNA-HGBC failed to induce SET expression. (Fig. 6f, g and Additional file 3: Figure S6G). In addition, the expression pattern of SET was similar to the expression of lncRNA-HGBC in the four GBC cell lines (Additional file 3: Figure S6H). Consistent with these data, IHC analysis revealed that SET expression was decreased in lncRNA-HGBC-depleted xenograft tumors, but increased in lncRNA-HGBC-overexpressing xenograft tumors, compared with the control group (Additional file 3: Figure S6I), suggesting that there was a co-expression relationship between lncRNA-HGBC and SET. In summary, these data strongly suggest that lncRNA-HGBC regulates SET expression by competitively binding to and inhibiting miR-502-3p.

### AKT is the downstream effector of SET and relationship of lncRNA with, miR-502-3p, SET and p-AKT in GBC

**Fig. 7:**
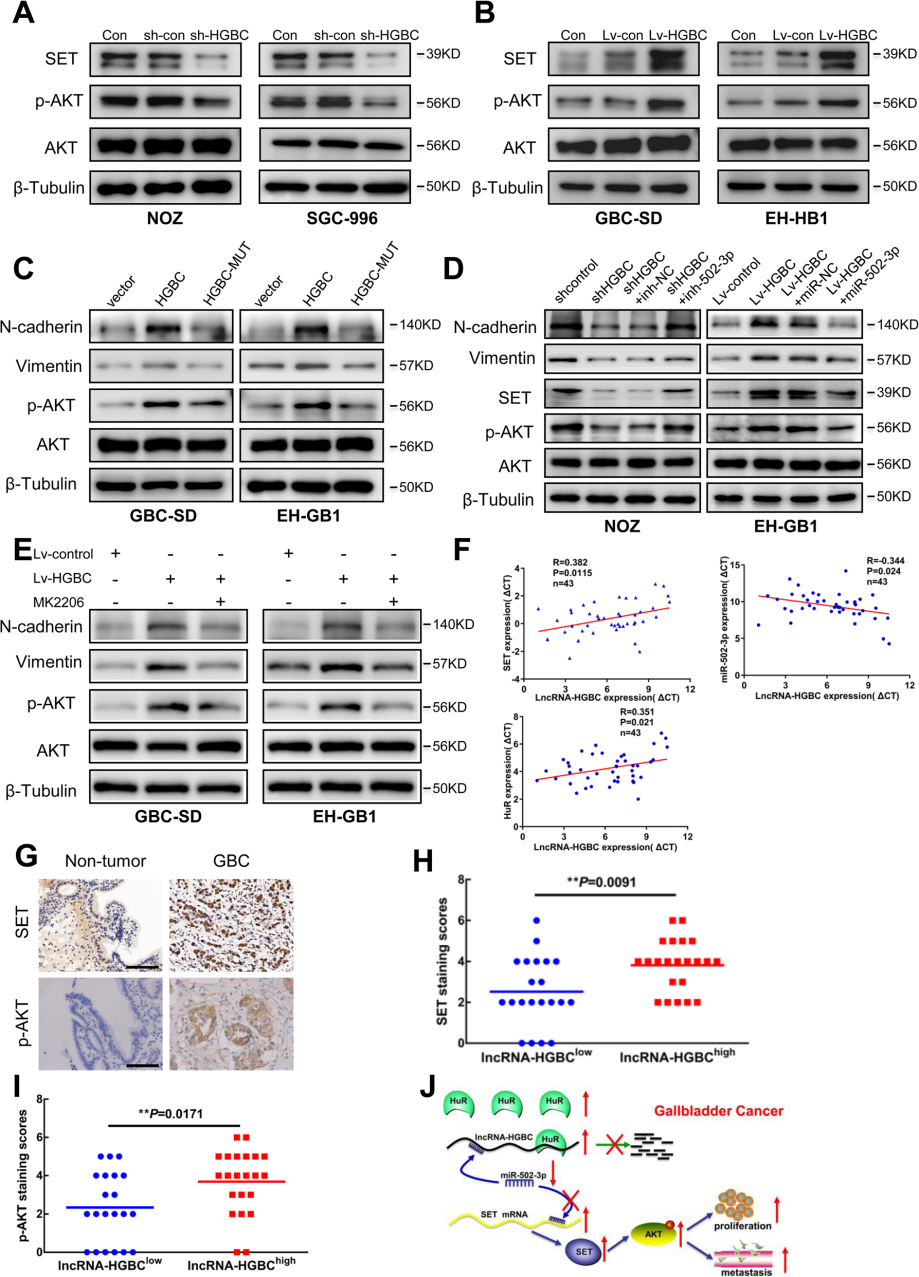
AKT is the downstream effector of SET and relationship of lncRNA with, miR-502-3p, SET and p-AKT in GBC. **a** Western blot assays of SET, p-AKT and AKT expression in shcontrol- or sh-HGBC-expressing NOZ or SGC-996 cells. **b** Western blot assays of SET, p-AKT and AKT expression in pcDNA3.1-based vector control and lncRNA-HGBC-expressing GBC-SD and EH-GB1 cells. **c** Western blotting analysis of N-cadherin, Vimentin, p-AKT and AKT expression in GBC-SD (left) and EH-GB1 (right) cells. **d** Western blotting analysis of N-cadherin, Vimentin, SET, p-AKT and AKT expression in indicated cells. **e** The expression of N-cadherin, vimentin, phosphorylated AKT and AKT was determined in GBC-SD and EH-GB1 cells in presence or absence of 10 uM MK2206. tubulin was used as the loading control. **f** The correlation between lncRNA-HGBC and SET (upper left), miR-502-3p (upper right) or HuR (bottom) expression was detected in 43 GBC specimens by qRT–PCR. The ΔCt values were subjected to Pearson correlation analysis. **g** Representative images of SET (top) or p-AKT (bottom) expression by immunohistochemical staining from non-tumor tissues and GBC tumors. Scale bars, 100 μm. **h-i** Scatterplots of the average staining scores for SET (h) or p-AKT (i) expression in GBC patients with low or high expression of lncRNA-HBC. **j** Schematic diagram of lncRNA-HGBC functions to promote tumor growth and metastasis in GBC cells

We next examined the potential molecular mechanisms of miR-502-3p and SET involved in GBC metastasis. As shown in Additional file 3: Figure S7A-S7E, miR-502-3p strongly inhibited the proliferation, colony formation and invasion capabilities of all of four GBC cell lines. Agreed with these data, direct knockdown of SET dramatically inhibited GBC cell proliferation and invasion (Additional file 3: Figure S8A-S8D). There is accumulating research evidence demonstrating that SET-induced oncogenic activity in various cancers is dependent on activation of AKT [36, 37]. Thus, it is reasonable to postulate that the axis of lncRNA-HGBC-miR-502-3p-SET-AKT acts as a pivotal system to mediate GBC development and metastasis. To approve this activated signaling in our system, we first examined AKT activation. Western blot assays showed that lncRNA-HGBC knockdown in NOZ and SGC-996 cells significantly inhibited the activated AKT (Fig. 7a); however, overexpression of lncRNA-HGBC induced phosphorylation of AKT in GBC-SD and EH-GB1 cells (Fig. 7b).

To determine if the lncRNA-HGBC-induced AKT activation is dependent on participation of miR-502-3p, we used miR-502-3p binding mutation of lncRNA-HGBC and found that lncRNA-HGBC overexpression in GSC-SD and EH-GB1 cells led to increases in AKT phosphorylation, N-cadherin, and vimentin, whereas lncRNA-HGBC MUT failed to induce these protein levels (Fig. 7c). In addition, introduction of miR-502-3p to lncRNA-HGBC-overexpressing EH-GB1 cells decreased these protein expressions; however, inhibition of miR-502-3p restored these levels that were inhibited by shHGBC in NOZ cells (Fig. 7d). AKT inhibitor MK2206 reversed effects of lncRNA-HGBC on expression of N-cadherin and vimentin (Fig. 7e), suggesting the tumor promoting role of lncRNA-HGBC requires AKT activation.

Finally, to validate the imitate association of individual factors in the lncRNA-HGBC-miR-502-3p-SET-AKT axis in GBC, we analyzed their correlations in 43 pairs of GBC tissue. LncRNA-HGBC was inversely correlated with miR-502-3p, whereas positively correlated with SET and HuR mRNA levels (Fig. 7f). HuR was upregulated in GBC tissues in comparison to non-tumor tissues (Additional file 3: Figure S9). IHC analysis of SET or p-AKT expression showed that both levels were significantly upregulated in GBC specimens compared with those in the non-tumorous tissues (Fig. 7g). Moreover, lncRNA-HGBC expression was positively correlated with SET and p-AKT expression (Fig. 7h, i). Collectively, all of these observations indicate that lncRNA-HGBC functions to sequester miR-502-3p then to activate SET and AKT downstream pathway, thus rendering tumor cells highly aggressive.

## Discussion

The human genome is pervasively transcribed to give rise to more than 80% non-coding genes in which lncRNAs constitute the primary elements, while only about 2% of genes encode for proteins. A wealth of research evidence has established the paradigm that lncRNAs are biologically functional molecules, in sharp contrast to initially accepted transcriptional “noise” [14, 38]. Given the cellular distribution of lncRNAs in both nucleus and cytoplasm, lncRNAs utilize a variety of molecular mechanisms to regulate gene activity and protein function, as some of lncRNAs participate in transcriptional interference, RNA splicing and miRNAquenching, while others can directly interact with transcriptional factors, hormone receptors and other RNA-binding proteins [15]. Thereof, it is not surprising to anticipate that deregulation of lncRNAs may result in multiple aspects of cellular dysfunction, even malignant transformation. In the current study, we discovered a novel cytosol lncRNA-HGBC with 1906 nt whose expression level was 16.6-fold higher in GBC than adjacent benign tissue and was correlated with positive lymph node metastasis and poorer patient survival, suggesting that lncRNA-HGBC may be a potential prognostic predictor or therapeutic target for GBC.

It is emerging that one of molecular mechanisms by which lncRNAs regulate gene expression is to interact with miRNA as ceRNAs that bind to MREs and protect miRNAs from binding to and repressing target RNAs [17, 39]. For example, lncRNA- MCM3AP-AS1 promotes tumor growth by specific binding to miR-194-5p, thus sequestering the miRNA and inducing FOXA1 expression [40]. In addition to the capability of MRE binding, lncRNAs display the strong ability to expedite degradation of miRNAs that inhibit target gene expression. lncRNA HOXA11-AS acts as a decoy for miR-1297, resulting in elevated expression of EZH2 in gastric cancer [41]. In concert with these findings, our current study demonstrates that lncRNA-HGBC interacts with miR-502-3p that is a tumor suppressor in hepatocellular carcinoma [35]. In addition, overexpression or knockdown of lncRNA-HGBC can correspondingly alter miR-502-3p expression, but the latter was unable to alter the former expression, indicating that lncRNA-HGBC not only interacts with and removes miR-502-3p from target gene inhibition, but also suppresses miR-502-3p expression. The substantial mechanisms of lncRNA-HGBC for regulating miR-502-3p expression warrant further investigation. Of note, miR-502-3p was down-regulated in GBC tissues and acted as a tumor suppressor to inhibit a target gene SET that promotes tumorigenesis in hepatocellular carcinoma [36], lung cancer [42] and breast cancer [43]. Indeed, lncRNA-HGBC-induced SET expression is fully dependent on the reduced expression or activity of miR-502-3p, because miR-502-3p mimic or overexpression of miR-502-3p can ablate lncRNA-HGBC activity. SET is known to inhibit protein phosphatase 2A (PP2A) [44] that can augment dephosphorylation and inactivationof AKT [45]. Given the feature that lncRNA-HGBC binds to and sequesters multiple miRNAs (e.g. miR-618), not limited to miR-502-3p, it is worthwhile to add considerable effort to reveal additional miRNAs that potentially regulate SET and others during GBC metastasis. The cell proliferation induced by lncRNA-HGBC in cultured cells was not as noticeable as developed tumor in vivo. The inconsistence could be ascribed to the following reasons. First, we used CCK8 as an in vitro assay to determine cell proliferation. This assay may have sensitivity limit as it determines cell growth by an enzyme-mediated colorimetric assay, instead of measuring DNA synthesis with radioactive material. Second, the in vitro changes in cell proliferation are only for tumor cells grown in a well in the absence of other cells or factors. In contrast, the in vivo tumors involve tumor microenvironment including stromal cells, growth factors, extracellular matrix/proteins and vessels, not limited to tumor cells. For example, lncRNA-HGBC expressed in tumor cells induces other angiogenic factors to stimulate blood vessel development which in turn promotes tumor cell proliferation. Last, we only observed in vitro a few days which do not represent a long period of cell growth in animals. A long-time culture such as a few weeks or months may virtually exhibit a notable increase in cell proliferation. Nevertheless, our current in vitro proliferation showed the induction with statistical significance, supporting our hypothesis that lncRNA has tumor-promoting activity.

HuR was unexpectedly identified as a lncRNA-HGBC-interacting protein. There is a large body of evidence demonstrating that HuR, a RNA binding protein, stabilizes mRNAs by binding to conserved AU-rich elements (AREs) within 3’UTRs and preventing gene degradation [46]. Nevertheless, HuR has been reported to be highly elevated in a number of cancers, such as brain tumor [47] and colon cancer [48]. Intriguingly, we also found that HuR can stabilize lncRNA-HGBC via 1759–1906 nt motif, which constitutes a positive feedback to rigorously stimulate lncRNA-HGBC expression and enhance tumor cell invasiveness. Indeed, we unveiled that HuR expression was upregulated and positively correlated with lncRNA-HGBC expression in GBC tissue. Therefore, our study has provided additional novel mechanistic insight into HuR in the regulation of lncRNA that drives GBC progression.

EMT is the key step mediating malignant transformation of a broad spectrum of cancers, including GBC [49]. We found that lncRNA-HGBC-induced metastasis was also associated with tumor cell EMT. EMT is genetically characterized by loss of epithelial cell biomarkers such as E-cadherin, but acquisition of mesenchymal proteins including N-cadherin, vimentin, smooth alpha actin and Slug. In line with these genetically phenotypic alterations, GBC cells strongly expressed N-cadherin and vimentin, and increased aggressive activity (cell migration and invasion). However, we currently can not exclude the possibility that these mesenchyme-associated proteins acquired by invasive tumor cells may be directly or indirectly regulated by miR-502-3p, HuR or SET. Thereof, it should be quite interesting to decipher potentially mechanistic link between EMT genes and lncRNA-HGBC downstream effectors.

## Conclusions

In summary, we identified a novel lncRNA-HGBC, which is stabilized by HuR, and acts as an endogenous sponge of miR-502-3p to promote GBC cell proliferation and metastasis. Inhibition of miR-502-3p upregulates SET which activates AKT signaling, resulting in cancer progression (Fig. 7j). The present study demonstrates that lncRNA-HGBC/miR-502-3p/SET/AKT axis plays a crucial role in GBC progression, pointing to lncRNA-HGBC as a potential therapeutic target for GBC.

## Supplementary information

**Additional file 1: Table S1.** Sequence information used in this study. **Table S2.** Sequences of primers used for qRT-PCR in this study. **Table S3.** Primary antibodies used in this study. **Table S4.** Genes connected to lncRNA-HGBC. **Table S5.** The potential lncRNA-HGBC-interacting proteins identified by mass spectrometry between 30 and 40 KD. **Table S6.** Predicted lncRNA-HGBC-interacting miRNAs.

**Additional file 2.** Supplementary Methods.

**Additional file 3: Figure S1.** Identification of lncRNA-HGBC and its noncoding nature analysis. **Figure S2.** LncRNA-HGBC promotes GBC cell proliferation and tumor growth. **Figure S3.** LncRNA-HGBC promotes the invasive capacity of GBC cells. **Figure S4.** LncRNA-HGBC did not influence HuR expression. **Figure S5.** Effects of miR-502-3p on lncRNA-HGBC expression. **Figure S6.** SET is a direct target of miR-502-3p in GBC cells. **Figure S7.** miR-502-3p inhibits GBC cell proliferation and invasion. **Figure S8.** Knockdown of SET inhibits GBC cell proliferation and invasion. **Figure S9.** HuR is upregulated in GBC tissues.

## Data Availability

The datasets used and/or analyzed during the current study are available from the corresponding author on reasonable request.

## Abbreviations

AGO2: Argonaute 2
ARE: AU-rich element
ceRNA: competing endogenous RNA
EMT: Epithelial-mesenchymal transition
FISH: Fluorescence in situ hybridization
FOXA1: Forkhead box A1
GAPDH: Glyceraldehyde-3-phosphate dehydrogenase
GBC: Gallbladder carcinoma
HuR: Hu antigen R
IHC: Immunohistochemistry
lncRNA-HGBC: long non-coding RNA Highly expressed in gallbladder carcinoma
MRE: MiRNA response element
MW: Molecular weight
PCNA: Proliferating cell nuclear antigen
qRT-PCR: quantitative real-time PCR
RACE: Rapid amplification of the cDNA ends
RIP: RNA immunoprecipitation
SET: SET nuclear proto-oncogene
