# Correction to: LncRNA-HGBC stabilized by HuR promotes gallbladder cancer progression by regulating miR-502-3p/SET/AKT axis

**DOI:** 10.1186/s12943-021-01386-8

**Published:** 2021-08-28

**Authors:** Yun-ping Hu, Yun-peng Jin, Xiang-song Wu, Yang Yang, Yong-sheng Li, Huai-feng Li, Shan-shan Xiang, Xiao-ling Song, Lin Jiang, Yi-jian Zhang, Wen Huang, Shi-li Chen, Fa-tao Liu, Chen Chen, Qin Zhu, Hong-zhuan Chen, Rong Shao, Ying-bin Liu

**Affiliations:** 1 Department of General Surgery, Xinhua Hospital, Affiliated to Shanghai Jiao Tong University School of Medicine, Building 25, Room 513, 1665 Kongjiang Road, Shanghai, 200092 China; 2 Shanghai Key Laboratory of Biliary Tract Disease Research, 1665 Kongjiang Road, Shanghai, 200092 China; 3 Shanghai Research Center of Biliary Tract Disease, 1665 Kongjiang Road, Shanghai, 200092 China; 4 Department of Pharmacology, Shanghai Jiao Tong University School of Medicine, W. Building 3, Room 407, 280 Chongqi Road, Shanghai, 200025 China

**Correction to: Mol Cancer 18, 167 (2019)**

**https://doi.org/10.1186/s12943-019-1097-9**

Following publication of the original article [1], the authors identified some minor errors in image-typesetting in Fig. 3; specifically in Fig. 3a (SGC-996 sh-control) and Fig. 3d (EH-GB1 Lv-control).

**Fig. 3:**
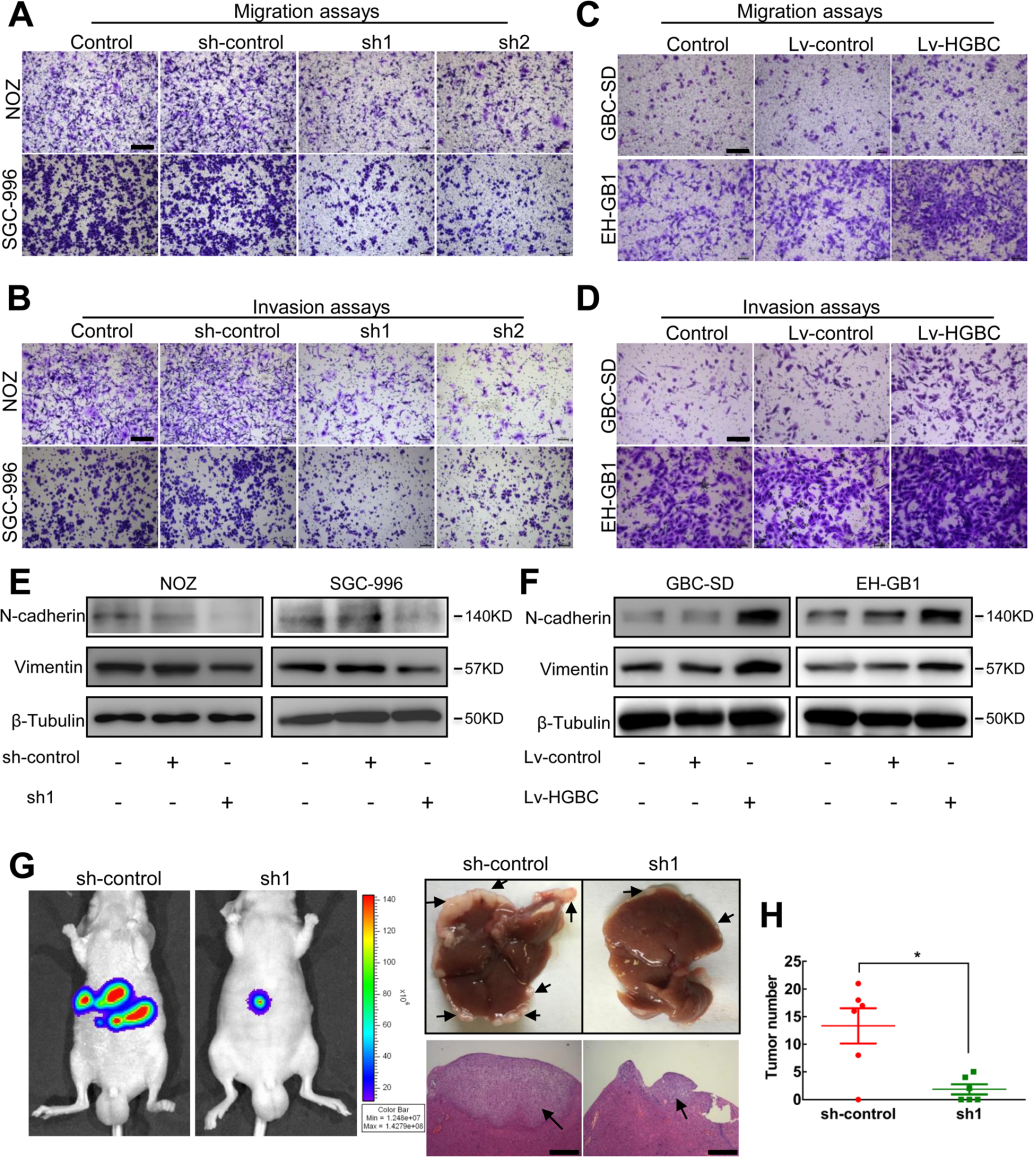
LncRNA-HGBC reinforces the invasive capacity of GBC cells. **a**, **b** Transwell assays (**a**) and Invasion assays (**b**) were used in NOZ and SGC-996 cells. Scale bars, 200 μm. **c**, **d** Transwell assays (**c**) and Invasion assays (**d**) were used in lncRNA-HGBC-overexpressing GBC-SD and EH-GB1 cells. Scale bars, 200 μm. **e**, **f** The protein levels of N-cadherin and Vimentin in control and lncRNA-HGBC-knockdown NOZ (left) or SGC-996 (right) cells, and in control and lncRNA-HGBC-overexpressing GBC-SD (left) or EH-GB1 (right) cells. **g** Representative images of luciferase signals in mice at the 6 weeks after intrasplenic injection with NOZ cell clones (left). Representative livers were shown and the isolated liver tissues sections were stained by hematoxylin and eosin (righ). Arrows indicate the metastasis nodules. Scale bars, 500 μm. **h** The average number of liver metastases in the intrasplenic injection model. Data are presented as mean ± SD of three independent experiments. *P < 0.05, **P < 0.01, ***P < 0.001

The corrected figure is given here. The correction does not have any effect on the results or conclusions of the paper.

The original article has been updated.
