# Supplementary material for: LncRNA-HGBC stabilized by HuR promotes gallbladder cancer progression by regulating miR-502-3p/SET/AKT axis
Source: Mol Cancer. 2019 Nov 21;18:167. doi: 10.1186/s12943-019-1097-9 (PMC6868746; doi:10.1186/s12943-019-1097-9)
Supplement: Supplementary file 2 — Additional file 2. Supplementary Methods. [file 12943_2019_1097_MOESM2_ESM.docx]

**Supplementary Methods**

**Cell culture and reagents**

GBC-SD cell line was purchased from Shanghai Institute for Biological Science, Chinese Academy of Science (Shanghai, China). NOZ were obtained from the Health Science Research Resources Bank (Osaka, Japan). SGC-996 was obtained from the Medical School at Tongji University (Shanghai, China). GBC-SD and EH-GB1 cells were grown in high-glucose Dulbecco’s modiﬁed Eagle medium (Gibco, Grand Island, NY, USA), NOZ was cultured in Williams (Gbico) and SGC-996 was maintained in RPMI 1640 (Hyclone, Logan, TX, USA) supplemented with 10% fetal bovine serum (Gibco). All cells were grown at 37°C and 5% CO_2_ in a humidiﬁed incubator (Thermo Fisher Scientific, USA). All the cell lines were verified by Short Tandem Repeat (STR) analysis.

NOZ and SGC-996 cells treated with or without HuR siRNA were incubated with α-aminitin (50 µM) (MedChem Express, New Jersey, USA) for indicated time. MK2206 was purchased from MedChem Express (New Jersey, USA), and incubated with indicated cells for 10 μM.

**Quantitative Real-Time PCR (qRT-PCR)**

Total RNA was extracted from cultured cells or tissue samples with TRIzol reagent (Invitrogen). The first strand cDNA was synthesized using a reverse transcription reagent kit (TaKaRa, Dalian, China) according to the manufacturer's instructions. qRT-PCR was performed using the SYBR-Green method with the specific primers provided in Additional file 1: Table S2. GAPDH was served as an endogenous control. The relative levels of genes were normalized using the 2^−ΔΔCt^ method. Each sample was tested from three independent experiments.

**Plasmids Construction**

The full length of lncRNA-HGBC or lncRNA-HGBC with mutation in the miR-502-3p binding sites was synthesized by GenScript (Nanjing, China) and subcloned into the Hind III and EcoR I sites of pcDNA3.1(+) vector (Invitrogen), named pcDNA3.1-HGBC, pcDNA3.1-HGBC-MUT(502-3p) respectively. For luciferase reporter assays, pcDNA3.1-HGBC or pcDNA3.1-HGBC-MUT(502-3p) was double digested by Sac I and Xho I and the fragments were inserted into pmirGLO vector, named pmirGLO-HGBC or pmirGLO-HGBC-MUT(502-3p) respectively. The 3ʹ untranslated region (UTR) of SET was PCR-amplified and subcloned into the downstream of the firefly luciferase gene within the pmirGLO vector. To construct MS2-RIP vectors, pSL-MS2-12X (Addgene) was double digested with EcoR I and Xho I, and the MS2-12X fragment was cloned into pcDNA3.1, pcDNA3.1-HGBC and pcDNA3.1-HGBC-MUT(502-3p), named pcDNA3.1-MS2, pcDNA3.1-MS2-HGBC and pcDNA3.1-MS2-HGBC-MUT(502-3p). To construct RNA-pull down or *in vitro* translation plasmids, pcDNA3.1-HGBC and pcDNA3.1-HGBC-MUT(502-3p) were digested with Hind III and EcoR I and cloned into pBluescript II SK (+), named pBluescript II SK-HGBC and pBluescript II SK-HGBC-MUT(502-3p). The pBluescript II SK (+)-HGBC deletion fragments were synthesized by GenScript and then subcloned into pBluescript II SK(+) plasmid. All the constructs were confirmed by DNA sequencing.

**Establishment of Stable Cell Lines**

To construct cell lines stably expressing lncRNA-HGBC, the full length of lncRNA-HGBC was synthesized and subcloned to the lentiviral vector Ubi-MCS-SV40-EGFP-IRES-puromycin (Genechem, Shanghai, China). Recombinant lentiviruses expressing the lncRNA-HGBC gene(Lv-HGBC) or the empty vector(Lv-NC) were produced by GeneChem (Shanghai, China). NOZ and SGC-996 cells were infected with concentrated virus. 72 h later, 1 µg/ml puromycin (Sigma) was added for two-week selection. Then, total RNA was extracted and real-time PCR was conducted to validate the overexpression of lncRNA-HGBC.

For the cell lines with stable knockdown of lncRNA-HGBC, three shRNA sequences targeting lncRNA-HGBC and negative control shRNA(shNC) were synthesized and cloned into hU6-MCS-Ubiquitin-EGFP-IRES-puromycin lentiviral vector. Then, the lentiviruses were produced in 293T cells. GBC-SD and EH-GB1 cells were infected with shHGBC or shNC virus. Cells were then selected for 2 weeks using puromycin. Of the three shHGBC viruses, the top two efficient shRNAs were used for the following functional assays. The shRNA sequences used in this study were listed in Additional file 1: Table S1.

**Transient Transfection**

For transient transfection, siRNAs, miRNA mimics and inhibitors and their respective negative control RNAs were synthesized (Biotend, Shanghai, China) and transfected into cells using the Lipofectamine 2000 kit (Invitrogen) according to the manufacturer’s instructions. For the plasmid transfection, 2 µg plasmids per each well of 6-well plate were transfected with Viafect (Promega). The cells were harvested at 48 h after transfection. The siRNA sequences were listed in Additional file 1: Table S1.

**Western Blot Analysis**

GBC cells were washed twice with ice-cold PBS and lysed on ice in RIPA buffer (Beyotime, Shanghai, China) containing Complete Mini Protease Inhibitor Cocktail and Phosphostop (Roche, Mannheim, Germany). Equal amount of protein samples were separated on a 10% polyacrylamide gel, then transferred onto a polyvinylidene fluoride (PVDF) membrane (Millipore, Darmstadt, Germany). After blocking with 5% skim milk for 1 h at RT, the membrane was incubated with indicated primary antibodies at 4 °C overnight followed by incubation with a goat anti- rabbit/anti-mouse-peroxidase-conjugated second antibody (1:5000, Abcam, Cambridge, UK). The signals of the membrane were visualized using an enhanced chemiluminescent (ECL) detection reagent (Rockford, IL, USA). The primary antibodies were listed in Additional file 1: Table S3.

**Immunohistochemistry assay**

Immunohistochemistry was performed on paraffin-embedded sections using a primary antibody against PCNA (Cell Signaling Technology), HuR (Abcam) or p-AKT (Cell Signaling Technology), SET (Abcam) followed by incubation with a goat anti-rabbit-peroxidase-conjugated second antibody (Santa Cruz). Then, DAB (3,3-diaminodbenzidine) substrate (Dako, Carpinteria, CA, USA) was added. HuR p-AKT and SET expression in GBC tissues was semi-quantitatively evaluated [1]. Briefly, the samples were classified into 3 groups based on the score of immunoreactions and the staining intensity: negative/weak (0–2 points), moderate (3–4 points) and strong (5–6 points) of staining.

**In Vitro Transwell Cell Migration and Invasion Assays**

The migratory and invasive ability of GBC cells was evaluated using the Transwell system (Corning, USA) according to the manufacturer’s instructions. In brief, cells were trypsinized, counted and re-suspended in serum- free media. GBC-SD (3×10^4^), NOZ (2×10^4^), OCUG-1 (3×10^4^) cells re-suspended in serum-free media were seeded into the upper chamber of an 8-μm pore size insert without Matrigel (Corning, New York, USA) or with Matrigel (BD Biosciences, Franklin Lakes, New Jersey, USA) in the 24-well plate. The lower chambers were filled with 600 μl media containing 10% FBS. After 24 h and 48 h of incubation, cells on the bottom surface of the membrane were fixed and stained with 0.1% crystal violet dye. Subsequently, the cells on the upper surface of the inserts were removed by scraping with a cotton swab. Migration and invasion was assessed by counting the number of penetrated cells in five randomly fields. The experiments were performed in triplicates.

**Cell Proliferation Analysis**

The CCK-8 assay and the colony formation assay were performed to evaluate cell proliferation. Briefly, for CCK-8 assay, GBC cells (1×10^3^) in 100 μL medium were seeded into 96-well plates. 10 μL CCK-8 solution (Dojindo, Kumamoto, Japan) was added to each well and incubated at 37˚C for 2 h at indicated time point. Finally, the absorbance at 450 nm was measured using a microplate reader (Bio-TEK, Saxony, USA). For colony formation assay, cells (4×10^2^) were placed into 6-well plate and cultured for 2 weeks. Cell colonies were fixed with 4% PFA, and stained with 0.1% crystal violet dye. Then stained colonies were photographed and manually counted. The results represent the average of 3 independent experiments.

**In vivo studies**

The male BALB/c athymic nude mice (randomized to each group, 4–6 weeks old) were purchased from Shanghai Laboratory Animal Center of the Chinese Academy of Sciences. All mice were housed under specific pathogen-free (SPF) conditions. All the animal experiments were conducted strictly according to the Guide for the Care and Use of Laboratory Animals, and approved by the Animal Care and Use Committee of the Xinhua Hospital.

Subcutaneous xenograft study was performed as previously described [2]. Briefly, A total of 1×10^6^ NOZ cells stably expressing shHGBC or negative control (shHGBC or shNC) or 1×10^6^ GBC-SD cells stably expressing lncRNA-HGBC or control vector (Lv-HGBC or Lv-NC) were subcutaneously injected into the right axilla of 4-week-old nude mice (n=5 per group). Tumor length and width were measured every week and tumor volume was calculated using the following formula: volume = length × (width^2^)/2. After 4 weeks, the mice were sacrificed and tumors were harvested and weighed. Additionally, part of the tumors was fixed and used for subsequent IHC analysis.

The intrasplenic injection model for *in vivo* metastasis assays was conducted as previously described [3]. Briefly, 2×10^6^ NOZ cells stably expressing both shHGBC or shNC and luciferase were injected into the spleen, then the spleen was oppressed for 2 min followed splenectomy. After 5 weeks, the metastases were monitored using the IVIS@ Lumina II system (Caliper Life Sciences, Hopkinton, MA) 10 min after intraperitoneal injection of 4.0 mg of luciferin (Promega) in 50μl of saline.

**Supplementary Reference:**

1. Shao R, Hamel K, Petersen L, Cao QJ, Arenas RB, Bigelow C*, et al.* YKL-40, a secreted glycoprotein, promotes tumor angiogenesis. Oncogene*.* 2009; 28: 4456-4468.

2. Hu YP, Wu ZB, Jiang L, Jin YP, Li HF, Zhang YJ*, et al.* STYK1 promotes cancer cell proliferation and malignant transformation by activating PI3K-AKT pathway in gallbladder carcinoma. Int J Biochem Cell Biol. 2018; 97: 16-27.

3. Wu XS, Wang F, Li HF, Hu YP, Jiang L, Zhang F*, et al.* LncRNA-PAGBC acts as a microRNA sponge and promotes gallbladder tumorigenesis. EMBO Rep. 2017; 18: 1837-1853.
