# Supplementary material for: LncRNA-HGBC stabilized by HuR promotes gallbladder cancer progression by regulating miR-502-3p/SET/AKT axis
Source: Mol Cancer. 2019 Nov 21;18:167. doi: 10.1186/s12943-019-1097-9 (PMC6868746; doi:10.1186/s12943-019-1097-9)
Supplement: Supplementary file 3 — Additional file 3: Figure S1. Identification of lncRNA-HGBC and its noncoding nature analysis. Figure S2. LncRNA-HGBC promotes GBC cell proliferation and tumor growth. Figure S3. LncRNA-HGBC promotes the invasive capacity of GBC cells. Figure S4. LncRNA-HGBC did not influence HuR expression. Figure S5. Effects of miR-502-3p on lncRNA-HGBC expression. Figure S6. SET is a direct target of miR-502-3p in GBC cells. Figure S7. miR-502-3p inhibits GBC cell proliferation and invasion. Figure S8. Knockdown of SET inhibits GBC cell proliferation and invasion. Figure S9. HuR is upregulated in GBC tissues. [file 12943_2019_1097_MOESM3_ESM.docx]

**Supplementary Figures**

**
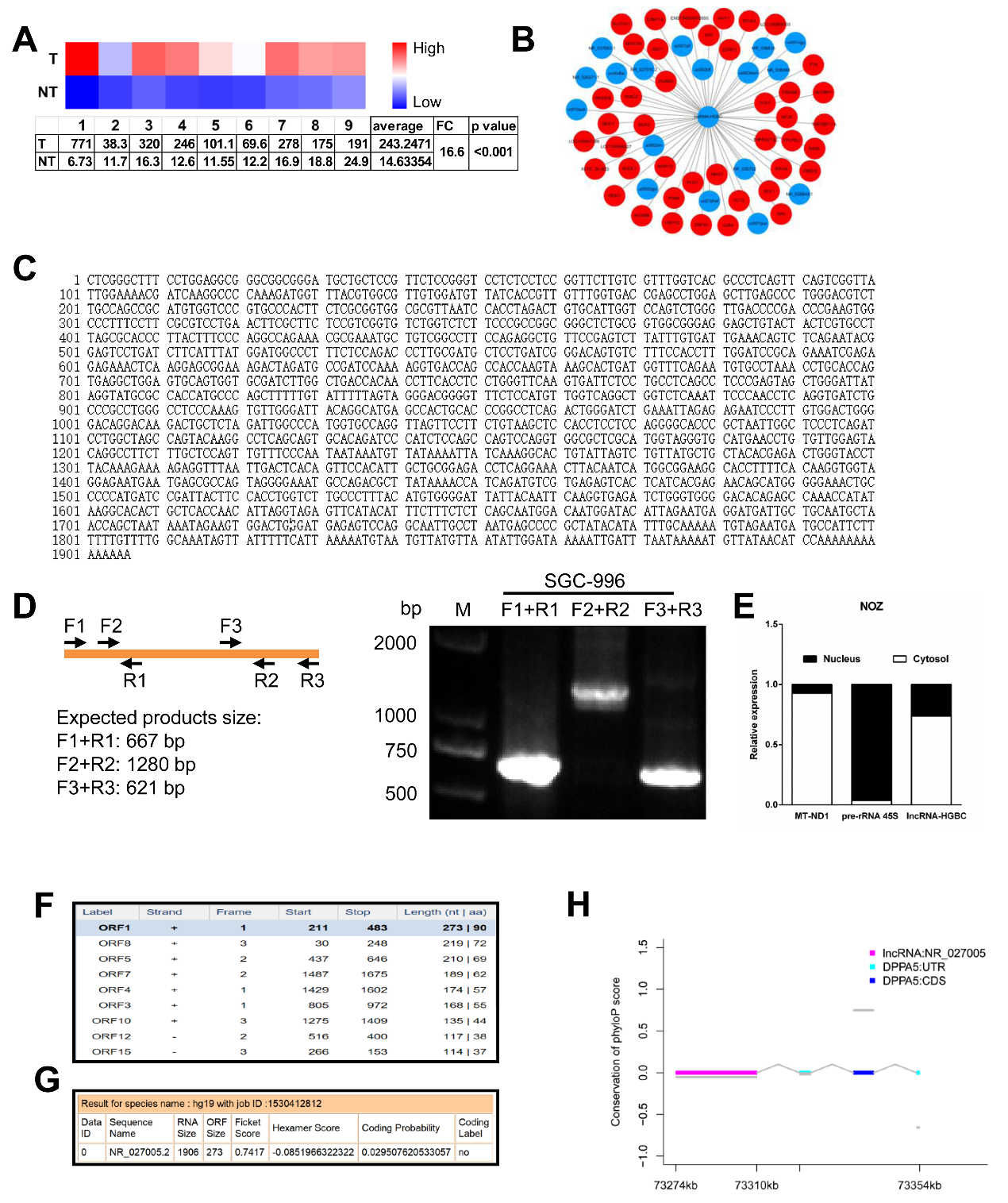
**

**Figure S1. Identification of lncRNA-HGBC and its noncoding nature analysis. A** LncRNA-HGBC expression was analyzed from our previous lncRNA microarray data performed in 9 pairs of GBC tissues and adjacent non-tumor tissues. Red represents high expression and blue represents low expression. The expression values of each sample from microarray results are shown on the bottom. FC: fold change. **B** lncRNA-HGBC subnetwork contains 17 lncRNAs (blue) and 42 protein-coding genes (red). **C** The nucleotide sequence of full-length lncRNA-HGBC determined by RACE. **D** PCR amplification to confirm the full-length of lncRNA-HGBC using 3 pairs of fragmented primers. **E** The nuclear and cytoplasmic RNA fractions of NOZ cells were detected by qRT-PCR. Pre-45S rRNA was a marker for nucleus and MT-ND1 was a marker for cytoplasm. **F** Putative proteins encoded by lncRNA-HGBC as predicted using ORF Finder. **G** Coding potential of lncRNA-HGBC predicted by CPAT. **H** The codon substitution frequency scores (CSF) of lncRNA-HGBC.


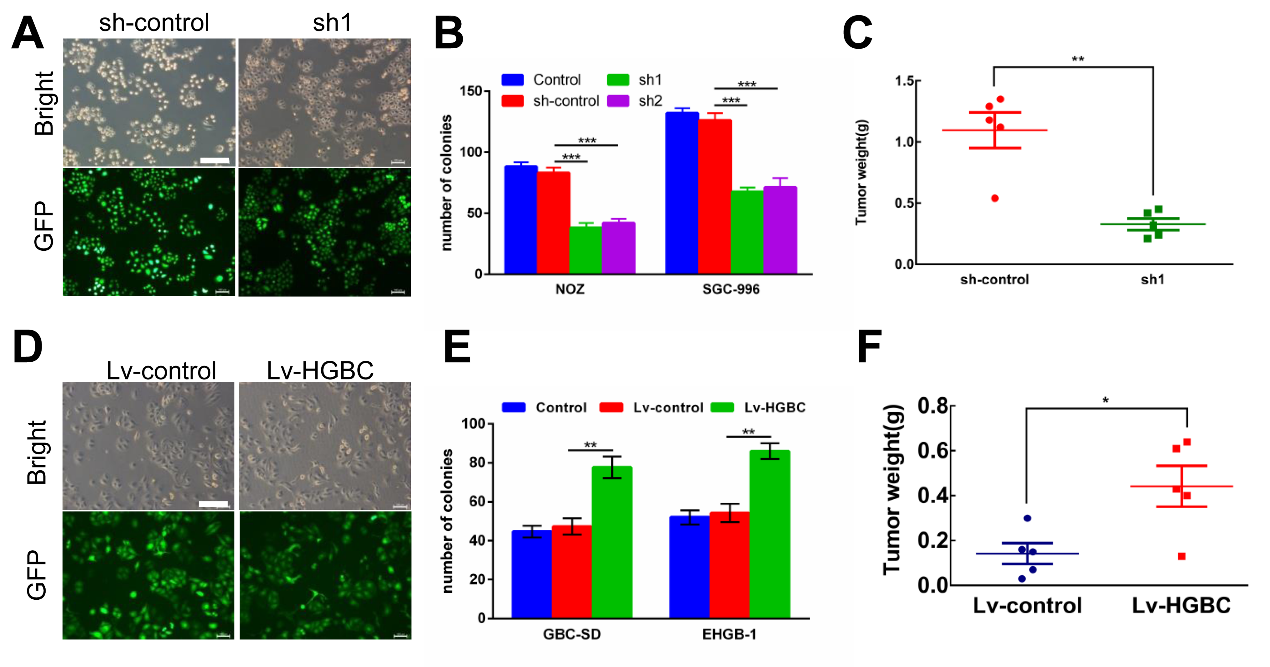


**Figure S2. LncRNA-HGBC promotes GBC cell proliferation and tumor growth. A** The GFP-labelled SGC-996 cells were photographed after incubation with the lentivirus. Scale bars, 200 μm. **B** The number of colonies formed by the indicated cells was statistically analysed. ****P* < 0.001. **C** Tumor weight was evaluated (***P*<0.01). **D** Transfection efficiency was determined by the GFP signal. Scale bars, 200 μm. **E** The colonies were statistically analysed. ***P* < 0.001. **F** Tumor weight was evaluated (**P*<0.05).

**
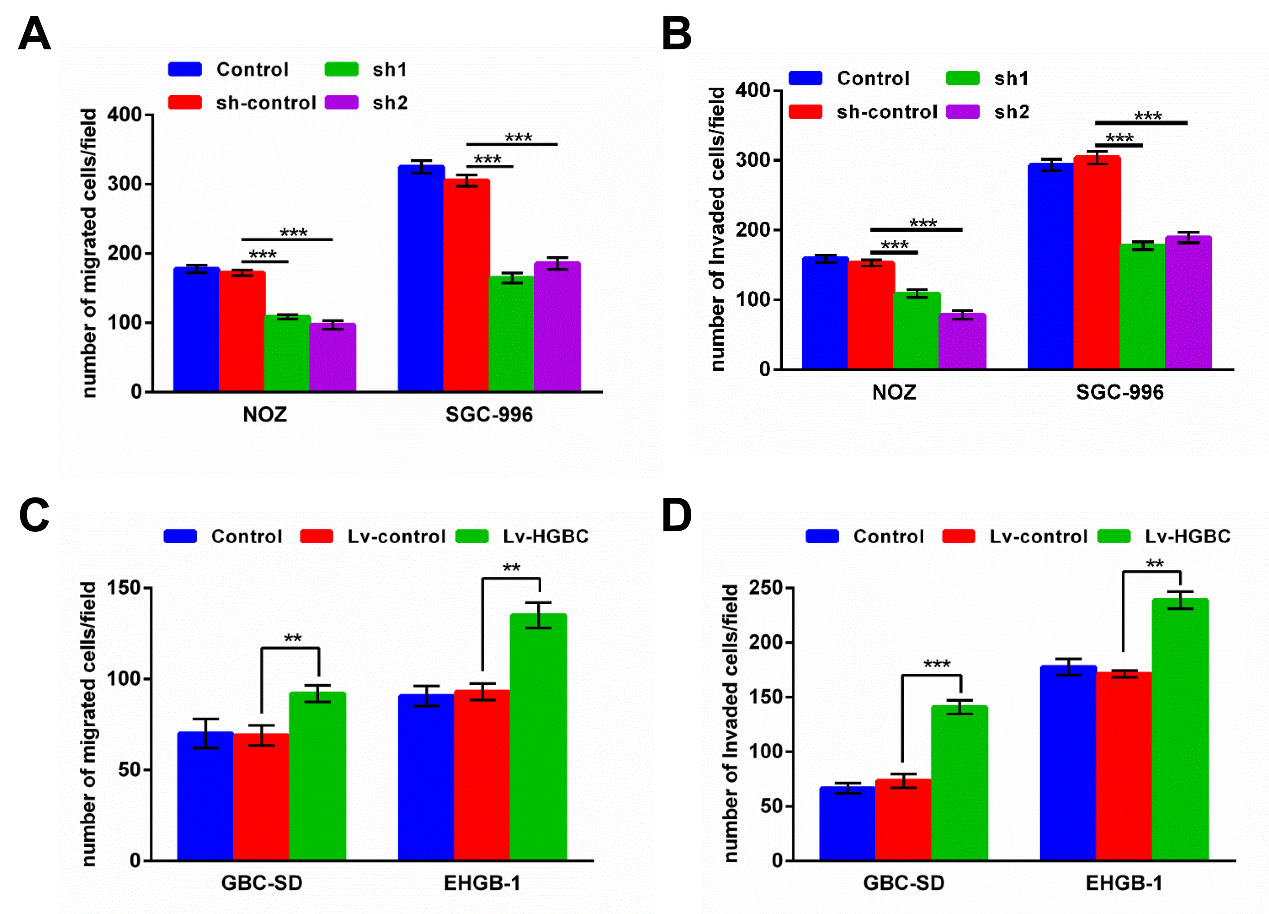
**

**Figure S3. LncRNA-HGBC promotes the invasive capacity of GBC cells.** **A, B** The number of migrated (**A**) or invaded (**B**) NOZ and SGC-996 cells with or without lncRNA-HGBC knockdown per field was counted. **C, D** The average number of migrated (**C**) or invaded (**D**) GBC-SD and EH-GB1 cells shown in Fig. 3c, d. The data are shown as the means ± SD of triplicate samples. *p < 0.05, **p < 0.01, ***p < 0.001.


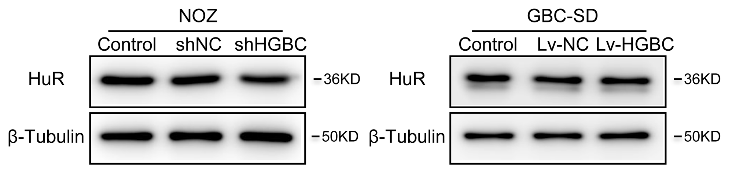


**Figure S4. LncRNA-HGBC did not influence HuR expression.** Western blot analysis of HuR protein levels after lncRNA-HGBC silence or overexpression.


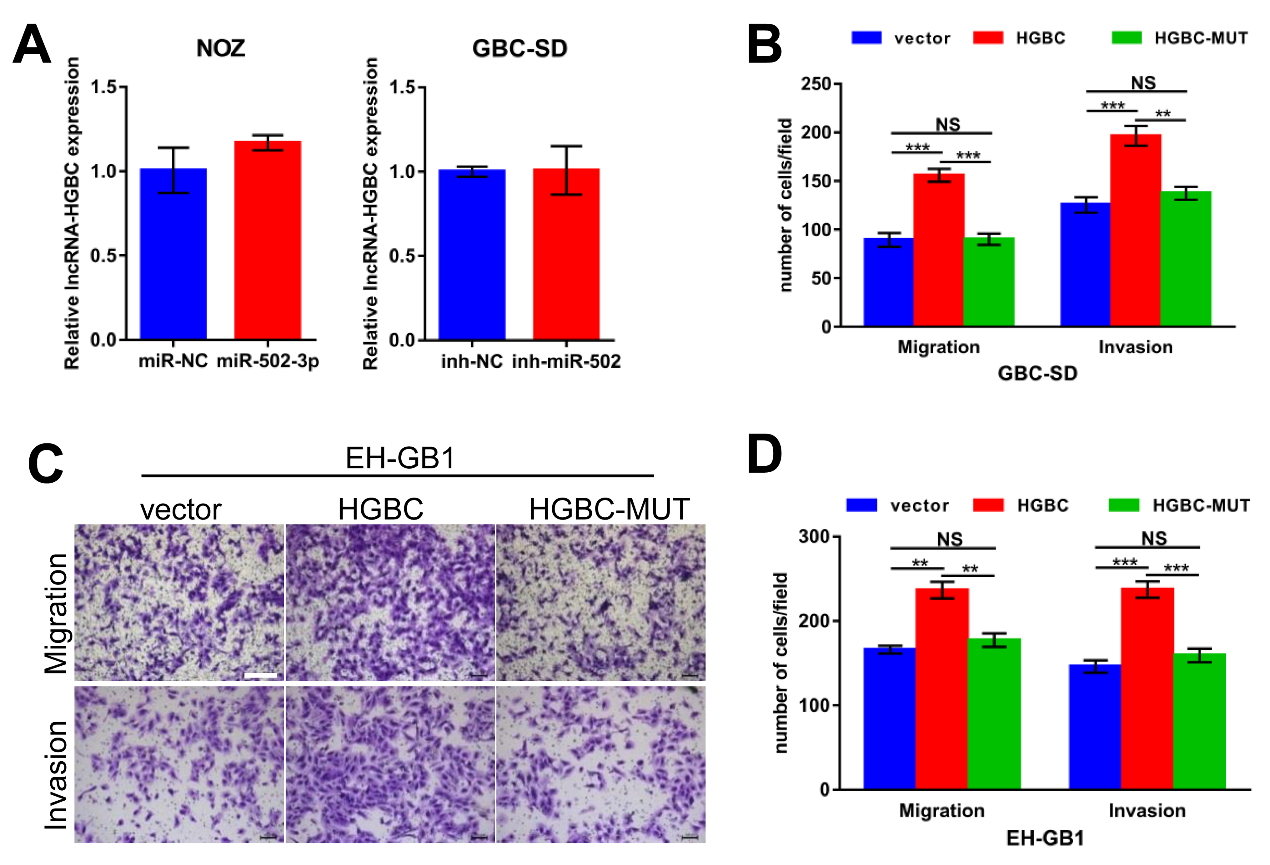


**Figure S5. Effects of miR-502-3p on lncRNA-HGBC expression.** **A** qRT-PCR analysis of lncRNA-HGBC expression in miR-502-3p-overexpressing NOZ cells (left) or miR-502-3p-depeleting GBC-SD cells (right). **B** The average number of migrated and invaded GBC-SD cells were shown. **C** Transwell migration (top) and invasion (bottom) assays of EH-GB1 cells. Scale bars, 200 μm. **D** The average number of migrated and invaded EH-GB1 cells were shown. **P* < 0.05, ***P* < 0.01, ****P* < 0.001 (Student’s t test).


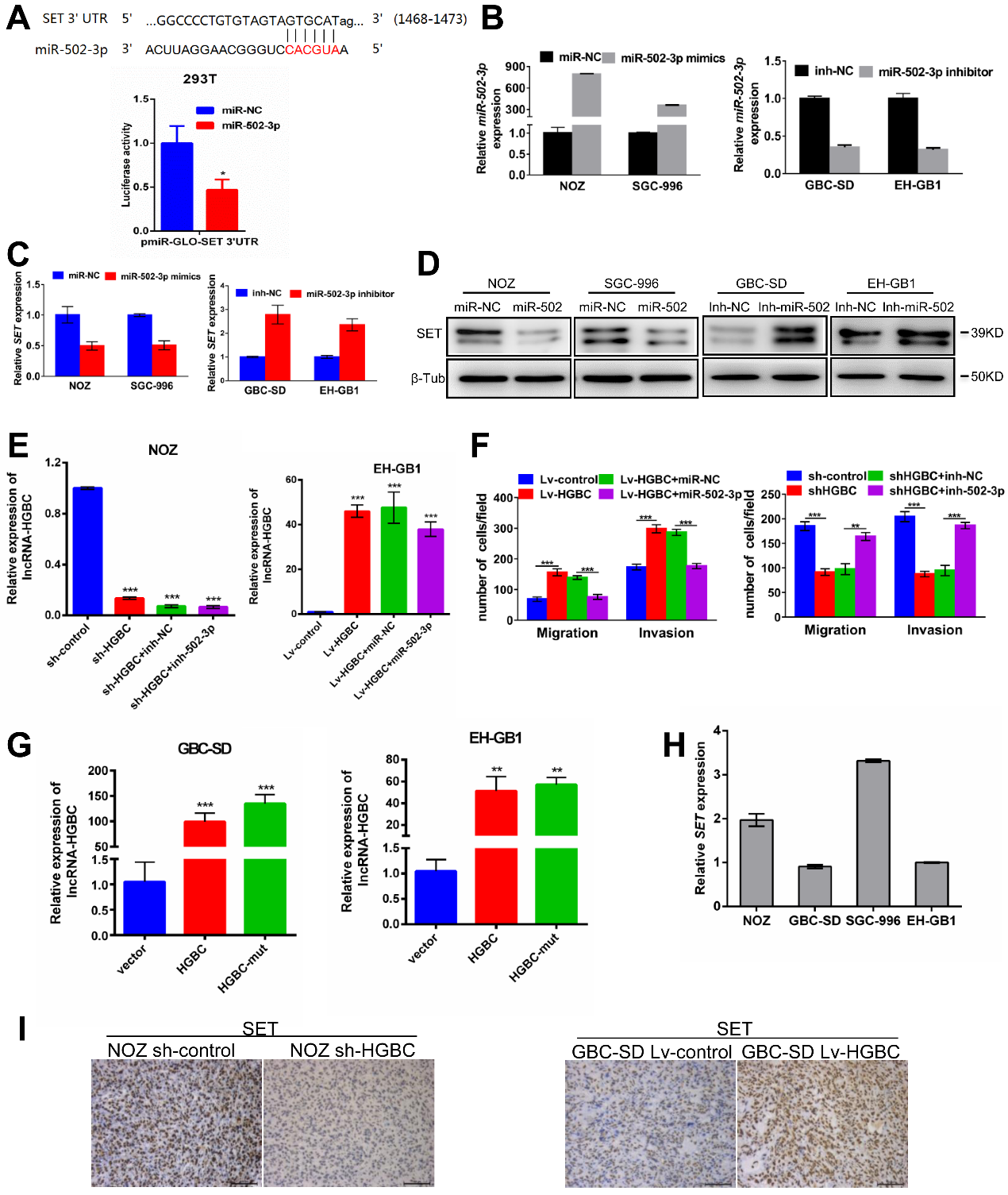


**Figure S6. SET is a direct target of miR-502-3p in GBC cells.** **A** Top: Putative miR-502-3p-binding sequence in the SET 3’ UTR. Bottom: Dual luciferase reporter assays in 293T cells revealed that miR-502-3p directly bound to the 3’-UTR region of SET. Firefly luciferase activity was normalized to Renilla luciferase expression for each sample. miR-NC was set to 1.0 in each experiment. **B** qRT-PCR analysis of miR-502-3p expression after transfection of miR-502-3p mimics (NOZ cells) and inhibitors (GBC-SD cells). **C** qRT-PCR analysis of SET expression in GBC cells after miR-502-3p overexpression or inhibition. **D** Western blot analysis of SET protein expression in miR-502-3p-mimic- or anti-miR-502-3p-transfected GBC cells. **E** qRT-PCR analysis of lncRNA-HGBC expression after indicated transfections. **F** The average number of migrated and invaded NOZ and EH-GB1 cells shown in Fig. 6d and 6e. **G** qRT-PCR analysis of lncRNA-HGBC levels after transfection of the indicated plasmids into GBC-SD and EH-GB1 cells. **H** qRT-PCR analysis of SET expression in four GBC cell lines. **I** IHC assay of SET expression in lncRNA-HGBC-depleted NOZ xenograft tumors (left) or lncRNA-HGBC-overexpressed GBC-SD xenograft tumors (right). Scale bars, 100 μm.


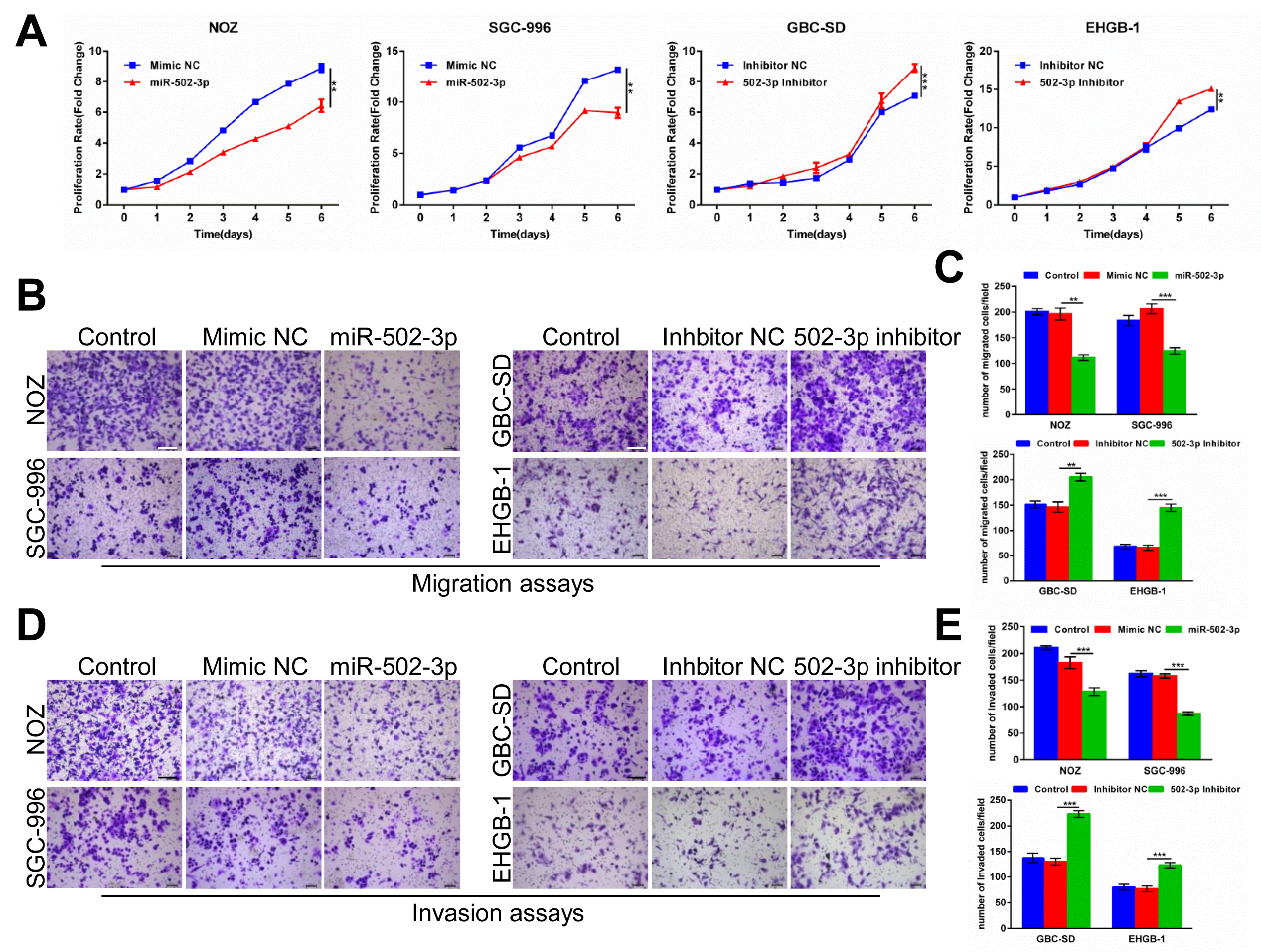


**Figure S7. miR-502-3p inhibits GBC cell proliferation and invasion.** **A-C** CCK-8 assays (**A**) and Transwell migration assays (**B, C**) of miR-502-3p-mimic- or anti-miR-502-3p-transfected GBC cells. Scale bars, 200 μm. **D, E** Invasion assays were performed in indicated cells with miR-502-3p (**D**) or anti-miR-502-3p (**E**) transfection. Scale bars, 200 μm. Data are presented as mean ± SD of three independent experiments. **P* < 0.05, ***P* < 0.01, ****P* < 0.001 (Student’s t test).


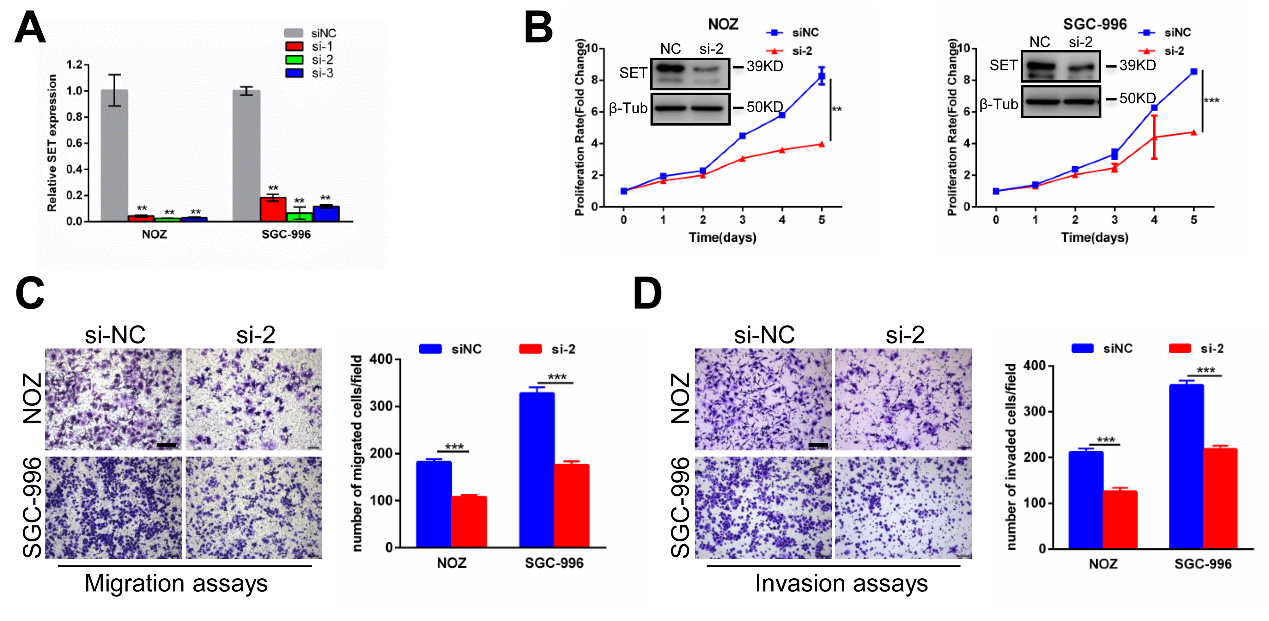


**Figure S8. Knockdown of SET inhibits GBC cell proliferation and invasion.** **A** SET expression level was measured in NOZ and SGC-996 cells by qRT–PCR after transfection of specific siRNAs targeting SET or a scrambled siRNA. **B** Cell proliferation rates were measured using cck-8 assay in NOZ (left) and SGC-996 (right) cells following SET silencing and western blot analysis showed siRNA targeting SET (si-2) reduced the SET expression level. **C** Transwell migration assays of SET-depleted NOZ or SGC-996 cells. Scale bars, 200 μm. **D** Invasion ability was examined after SET knockdown in NOZ and SGC-996 cells. Scale bars, 200 μm. Data are presented as mean ± SD of three independent experiments. **P* < 0.05, ***P* < 0.01, ****P* < 0.001 (Student’s t test).


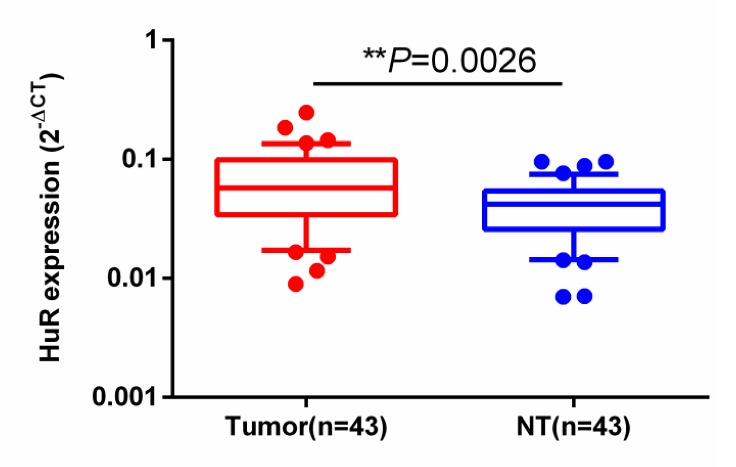


**Figure S9. HuR is upregulated in GBC tissues.** Box plots of the relative expression of HuR in 43 paired human GBC tissues and non-tumour tissues (NT).
